# Passive Sensing for Mental Health Monitoring Using Machine Learning With Wearables and Smartphones: Scoping Review

**DOI:** 10.2196/77066

**Published:** 2025-08-14

**Authors:** ShiYing Shen, Wenhao Qi, Jianwen Zeng, Sixie Li, Xin Liu, Xiaohong Zhu, Chaoqun Dong, Bin Wang, Yankai Shi, Jiani Yao, Bingsheng Wang, Xiajing Lou, Simin Gu, Pan Li, Jinghua Wang, Guowei Jiang, Shihua Cao

**Affiliations:** 1 School of Nursing Hangzhou Normal University Hangzhou China; 2 School of Nursing Zhejiang Chinese Medical University Hangzhou China; 3 Centre for Medical Informatics Usher Institute University of Edinburgh Edinburgh United Kingdom; 4 Department of Neurology The Affiliated Hospital of Hangzhou Normal University Hangzhou China; 5 Department of Psychiatry and Neuropsychology and Alzheimer Center Limburg School for Mental Health and Neuroscience (MHeNS) Maastricht University Maastricht The Netherlands; 6 Key Engineering Research Center of Mobile Health Management System Hangzhou China

**Keywords:** passive sensing, mental health monitoring, mental disorders, machine learning, digital biomarkers, wearable devices, scoping review, artificial intelligence, AI

## Abstract

**Background:**

Mental health issues have become a significant global public health challenge. Traditional assessments rely on subjective methods with limited ecological validity. Passive sensing via wearable devices and smartphones, combined with machine learning (ML), enables objective, continuous, and noninvasive mental health monitoring.

**Objective:**

This study aimed to provide a comprehensive review of the current state of passive sensing–based and ML technologies for mental health monitoring. We summarized the technical approaches, revealed the association patterns between behavioral features and mental disorders, and explored potential directions for future advancements.

**Methods:**

This scoping review adhered to the PRISMA-ScR (Preferred Reporting Items for Systematic Reviews and Meta-Analyses extension for Scoping Reviews) guidelines and was prospectively registered on the Open Science Framework. We systematically searched 7 databases (Web of Science, PubMed, IEEE Xplore, Embase, PsycINFO, Scopus, and ACM Digital Library) for studies published between January 2015 and February 2025. We included 42 peer-reviewed studies that used passive sensing from wearables or smartphones with ML to monitor clinically diagnosed mental disorders, such as depression and anxiety. Data were synthesized across technical dimensions (data collection, preprocessing, feature engineering, and ML models) and clinical associations, with behavioral features categorized into 8 domains.

**Results:**

The 42 included studies were predominantly cohort designs (23/42, 55%), with a median sample size of 60.5 (IQR 54-99). Most studies focused on depression (23/42, 55%) and anxiety (9/42, 21%) using primarily wrist-worn devices (32/42, 76%) collecting heart rate (28/42, 67%), movement index (25/42, 60%), and step count (17/42, 40%) as key biomarkers. Deep learning models (eg, convolutional neural networks and long short-term memory) showed high accuracy, while traditional ML (eg, random forest) remained prevalent due to better interpretability. We identified critical limitations, including small samples (32/42, 76% with N<100), short monitoring periods (19/42, 45% <7 days), scarce external validation (1/42, 2%), and limited reporting on data anonymization (6/42, 14%).

**Conclusions:**

While passive sensing and ML demonstrate promising accuracy (eg, convolutional neural network–long short-term memory achieving 92.16% in anxiety detection), the evidence remains constrained by three key limitations: (1) methodological heterogeneity (32/42, 76% single-device studies; 19/42, 45% with <7-day monitoring), (2) high risk of bias from small samples (median 60.5, IQR 54-99 participants) and scarce external validation (1/42, 2%), and (3) ethical gaps (only 6/42, 14% addressing anonymization). These findings underscore the technology’s potential to transform mental health care through objective, continuous monitoring—particularly for depression (heart rate and step count biomarkers) and anxiety (sleep and social interaction patterns). However, clinical translation requires standardized protocols, larger longitudinal studies (≥3 months), and ethical frameworks for data privacy. Future work should prioritize multimodal sensor fusion and explainable artificial intelligence to bridge the gap between technical performance and clinical deployability.

## Introduction

### Background

Mental health issues have become a significant global public health challenge, affecting the quality of life and social functioning of hundreds of millions of people [1]. According to the World Health Organization, there are 322 million people with depression and 264 million people with anxiety worldwide, yet a large number of individuals still fail to receive timely diagnosis and intervention [2,3]. Traditional mental health assessments mainly rely on clinical interviews [4] and self-report scales [5], which, although widely used, have limitations such as high subjectivity [6], long assessment periods [7], and insufficient ecological validity [8], making it difficult to capture dynamic psychological changes in daily life [9]. In addition, many patients seek help only when symptoms become severe, hindering early intervention [10]. Therefore, there is an urgent need for an objective, continuous, and noninvasive mental health monitoring method to address the limitations of traditional approaches.

With the proliferation of smartphones, wearable devices, and other digital health technologies, passive sensing has emerged as a promising tool for mental health monitoring [11,12]. This technology uses embedded sensors such as accelerometers, GPS, and microphones to continuously and unobtrusively collect data on users’ behavior, physiology, and environment without requiring active participation [13]. These data reflect an individual’s daily activity patterns, social behaviors, voice features, and sleep quality, indirectly revealing their psychological state [14]. For example, GPS trajectories can be used to assess the level of social activity [14], accelerometer data can be used to analyze physical activity patterns [15], and voice analysis may detect emotional changes [16]. Compared to traditional methods, passive sensing offers significant advantages in terms of objectivity, continuity, and low intervention [12], showing great potential for monitoring and early warning of various mental disorders, including depression [17], anxiety [18], and bipolar disorder [19].

However, the data generated through passive sensing are typically high-dimensional, noisy, and unstructured, making it challenging to process using traditional data analysis methods [20]. The introduction of machine learning (ML) techniques provides a solution to this challenge. ML can automatically extract features from vast and complex sensor data and establish a mapping relationship between data and mental health status [11]. For instance, supervised learning algorithms (eg, support vector machine [SVM] and random forest) can be trained on labeled data to classify depression or anxiety levels [21,22], unsupervised learning (eg, clustering analysis) can uncover latent behavior patterns [23], and deep learning (eg, convolutional neural networks [CNNs] and recurrent neural networks) can extract deep features related to mental health directly from time-series data such as voice or motion sensors, overcoming the limitations of manual feature engineering and significantly improving recognition performance [24]. The continuous data collection by smart devices, combined with advanced algorithms, enables real-time monitoring and early warning of mental health status, providing strong data support for personalized interventions and precision medicine [25].

### Objectives

Although several reviews have addressed the application of passive sensing technologies in mental health monitoring, significant methodological gaps still exist. First, most reviews focus on the sensing technology itself rather than the integration of sensing with ML [26-28]. Second, there is limited discussion of key aspects of ML (such as data preprocessing and feature engineering), restricting the reproducibility and optimization potential of the technology [29-31]. Moreover, existing studies often focus on a single mental disorder (eg, depression [32,33] or anxiety [34]). Furthermore, they fail to include relevant databases such as PsycINFO [34-36], IEEE Xplore [37], and the ACM Digital Library [34,38]. On the basis of these gaps, this study aimed to conduct a scoping review to comprehensively assess the current application and development trends of ML techniques in mental health monitoring based on passive sensing data from wearable devices and smartphones. Specific objectives included to (1) synthesize and summarize current mainstream technical frameworks and commonly used methods for data acquisition, preprocessing, feature engineering, and algorithm selection; (2) evaluate association patterns between diverse behavioral features (eg, sleep, physical activity, and social interaction) and specific mental health conditions (eg, depression and anxiety); (3) identify the primary limitations and challenges of existing approaches with respect to sample representativeness, data quality, and model generalizability; and (4) explore potential future improvements in standardized data collection, algorithm optimization, and ethical guidelines.

## Methods

### Overview

This scoping review adhered to the PRISMA-ScR (Preferred Reporting Items for Systematic Reviews and Meta-Analyses extension for Scoping Reviews) [39] guidelines to ensure methodological rigor and transparency. The review protocol was preregistered on the Open Science Framework (registration 10.17605/OSF.IO/74ACP). The full PRISMA-ScR checklist is provided in Multimedia Appendix 1.

### Data Sources and Search Strategy

To ensure the systematic and reproducible nature of the literature search, we used an interdisciplinary database search strategy covering 7 authoritative databases across the fields of medicine, engineering, psychology, and computer science: Web of Science Core Collection, PubMed, IEEE Xplore, Embase, PsycINFO, Scopus, and the ACM Digital Library. In addition, we manually screened the reference lists of the included studies and used citation tracking to extend the search scope.

Before the official search, we collaborated with librarians, mental health experts, and health informatics specialists to develop a detailed search strategy based on the definitions in the Biomarkers, Endpoints, and Other Tools vocabulary [40]. The core keywords included “wearable devices,” “smartphones,” “mental health,” and “machine learning,” which were combined using Boolean logic. All search queries were pretested to ensure high relevance across different databases. To mitigate any bias introduced by daily updates to the databases, all searches were completed synchronously on February 3, 2025. After removing duplicates using the EndNote X20 software (Clarivate Analytics), the retrieved studies were systematically organized and analyzed. The specific search strategy is detailed in Multimedia Appendix 2.

### Inclusion and Exclusion Criteria

Study eligibility was determined using the population, intervention, comparison, outcomes, and study design framework, as shown in Textbox 1, with additional methodological considerations detailed later in this section.

**Eligibility criteria based on the population, intervention, comparison, outcomes, and study design framework.**

**Inclusion criteria**

- Population: participants with mental health–related disorders (≥70% of the sample)
- Intervention: passive data collection using wearable and smartphone sensors and machine learning–based prediction or detection of mental health conditions
- Outcomes: clinically diagnosed mental health disorders (eg, depression and anxiety) monitored via passive sensing data collected in naturalistic settings using wearable devices or smartphones
- Study design: peer-reviewed original research in English and published between 2015 and 2025

**Exclusion criteria**

- Population: studies focusing solely on transient states (eg, mood or stress) without clinical diagnoses and prototype devices (eg, electronic tattoos and textile sensors)
- Intervention: active data (eg, questionnaires) as model input features and simulated or laboratory-only task data
- Outcomes: prediction of isolated physiological signals (eg, heart rate) without mental health linkage
- Study design: nonresearch articles (reviews and protocols); unavailable full text or incomplete data; and conference papers, abstracts, or nonjournal publications

Two key methodological considerations guided our eligibility assessment. First, given the rapid evolution of wearable artificial intelligence (AI) technology and our focus on modern methodologies, we restricted the search to articles published after 2015. Second, while changes in mental health states (eg, emotions, mood, and stress) may serve as potential indicators of disorders such as depression or anxiety [41], examining these factors in isolation fails to reflect clinical diagnoses of mental health conditions [42,43]. Thus, we excluded studies focusing solely on these states to maintain research precision.

It should be noted that the comparison component of the population, intervention, comparison, outcomes, and study design was not applied as our scoping review focused on technological applications rather than therapeutic comparisons.

### Screening Strategy

Before the formal screening process, the evaluators (SYS and JZ) randomly selected 30 articles and conducted a preliminary review of titles and abstracts followed by full-text evaluation based on the inclusion and exclusion criteria. A double-blind cross-validation approach was used. The pilot screening yielded a Cohen κ of 0.91, demonstrating a high level of agreement between the evaluators, and no adjustments to the screening process or criteria were necessary. Subsequently, SYS and JZ independently screened the remaining articles, and any discrepancies were resolved through discussion with a third-party evaluator (WQ), who made the final decision. On February 15, 2025, the screening was completed, and 42 studies meeting the criteria were included.

### Data Extraction and Synthesis

To explore the application of ML methods in the field of mental health and analyze how passive, noninvasive sensor data combined with ML techniques can be used to predict, detect, or monitor mental health disorders and outcomes, we followed the process of ML model development and validation. This process clarified the key stages of data acquisition, data processing, ML method application, and health outcome screening.

The first stage related to study characteristics. We used a standardized form to extract the key characteristics of each included study. Two independent reviewers collected data on study design, sample size, participant demographics, and the specific mental health issues addressed. For gender data, raw values were converted into percentages, with median values and IQRs subsequently calculated. In addition, we documented the duration of data collection and the sources of funding for each study. Using the R packages *ggplot2*, *dplyr*, and *ggsci* (R Foundation for Statistical Computing), we visualized the distribution of sample characteristics and funding sources.

In terms of data acquisition, we focused on the measurement environment, types of devices used, and specific configuration parameters reported in the studies. Detailed information, including device brands, sensor types, and body placement, was systematically documented. Using R packages such as *ggplot2*, *tidyr*, *dplyr*, *igraph*, *fmsb*, and *tibble*, we constructed an integrated network diagram illustrating the relationships among device types, sensor categories, and behavioral features. In addition, *reshape2* and *ggplot2* were used to analyze feature use patterns across studies and generate a heat map highlighting the prevalence of specific features under different mental health conditions.

Regarding data preparation, we systematically compiled the data preprocessing techniques used across the included studies. This encompassed a variety of signal denoising methods, including specific filtering algorithms and their parameter settings; strategies for handling missing data; and approaches to address class imbalance. These approaches were organized into comparative tables to highlight methodological variations among research teams. Special emphasis was placed on sensor-specific preprocessing techniques tailored to different data modalities.

Regarding feature engineering, we extracted features along three dimensions: (1) time window parameters, including window length and overlap ratio; (2) feature extraction techniques, spanning time-domain and frequency-domain analyses; and (3) feature selection strategies. We also compiled methods for assessing feature importance and, across 31% (13/42) of the studies, extracted quantitative metrics such as importance rankings and effect sizes. Data were processed using the *tidyverse* package in R, and we visualized the top 3 features’ associations with psychiatric disorders in each study via network graphs rendered using *ggraph* and *igraph*.

In terms of ML methods, we comprehensively documented the ML strategies used across the studies, including algorithm classes, model validation schemes, and performance metrics. Using the R packages *dplyr*, *networkD3*, *htmlwidgets*, and *jsonlite*, we generated an overview diagram summarizing the application of ML methods.

**Figure 1:**
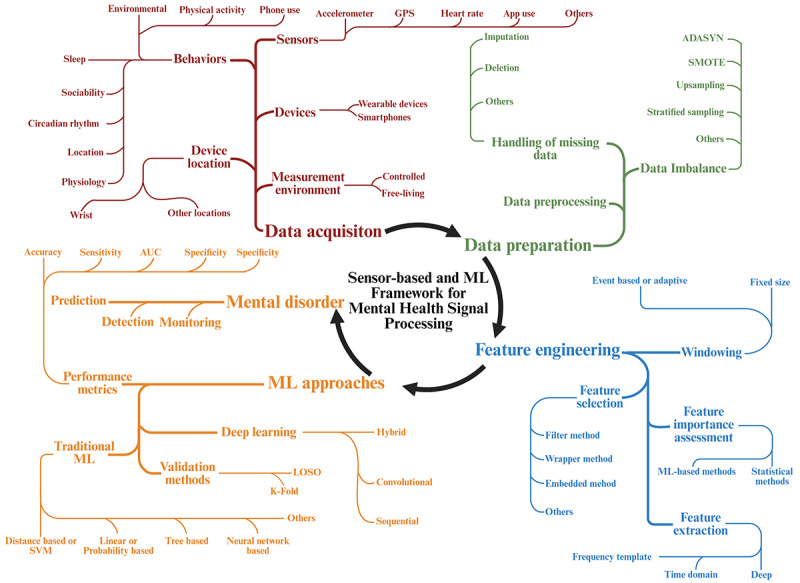
Overview of key data items extracted from the included studies. ADASYN: adaptive synthetic sampling; AUC: area under the curve; LOSO: leave one subject out; ML: machine learning; SMOTE: synthetic minority oversampling technique; SVM: support vector machine.

Figure 1 provides an overview of the key data items extracted from the included studies. In addition, we categorized the features into 8 main behavioral categories: sleep, physical activity, circadian rhythm, sociability, location, physiology, phone use, and environmental behavior [33]. Each behavioral category included several lower-level features that resulted from the integration of various individual features reported in each study. As a result, a single study may identify multiple associations with the same feature. Detailed definitions and classifications of these behavioral categories can be found in Multimedia Appendix 3.

## Results

### Study Characteristics

**Figure 2:**
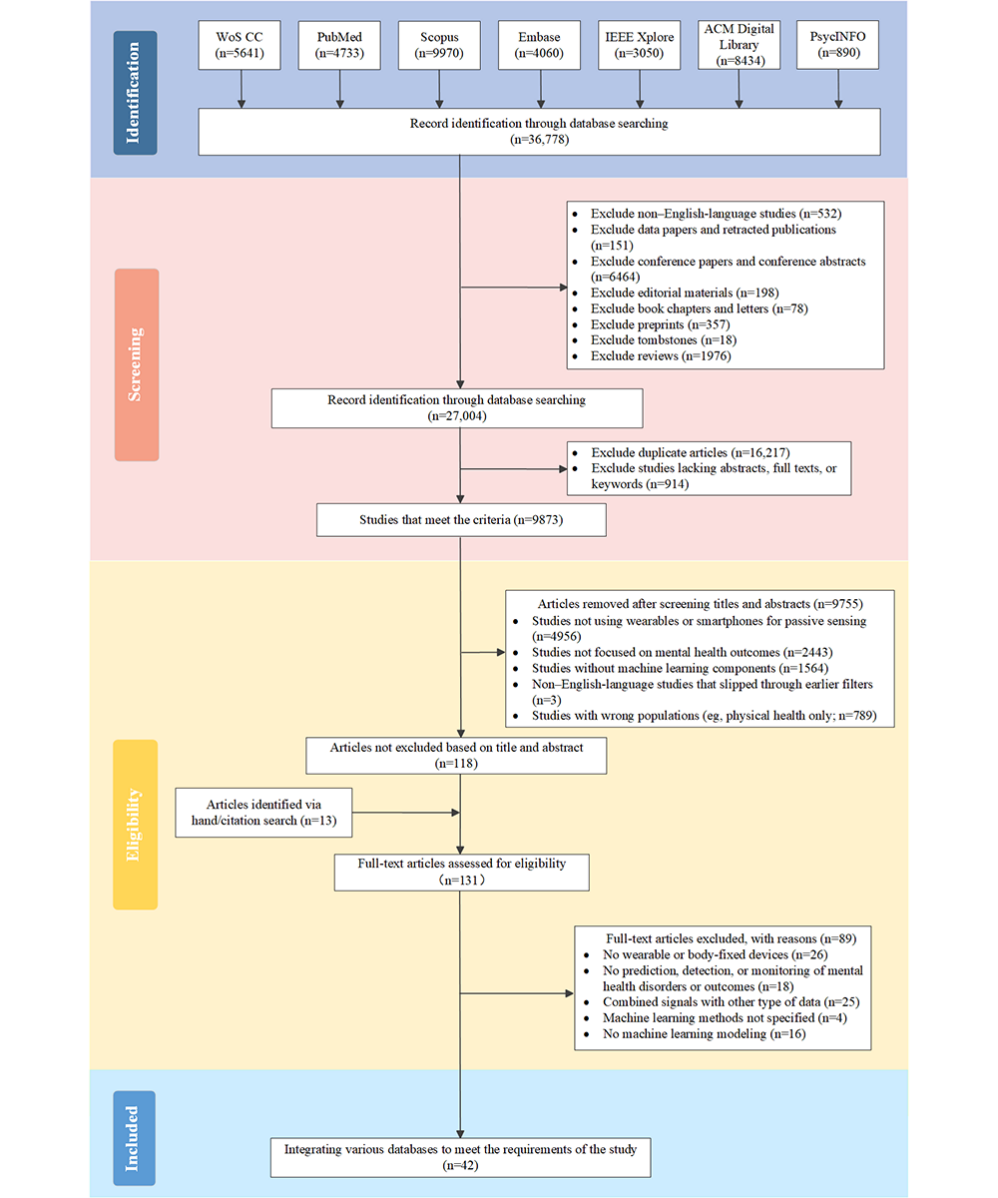
Search and filter process diagram. WoS CC: Web of Science Core Collection.

The study selection process is detailed in the PRISMA (Preferred Reporting Items for Systematic Reviews and Meta-Analyses) flowchart (Figure 2). A total of 42 studies were included in this scoping review. Among them, cohort studies accounted for the largest proportion (n=23, 55%), followed by cross-sectional studies (n=14, 33%). The median overall sample size was 60.5 (IQR 54-99) participants, whereas the median proportion of female participants was 57.5% (IQR 46.25%-67.34%). The study populations were primarily focused on depression (n=23, 55%) and anxiety disorders (n=9, 21%), followed by bipolar disorder (n=4, 10%) and schizophrenia (n=6, 14%).

In terms of control group design, 48% (20/42) of the studies used healthy or population-based control groups, 17% (7/42) compared patients with different mental health conditions, and only 2% (1/42) were based entirely on a healthy population.

**Figure 3:**
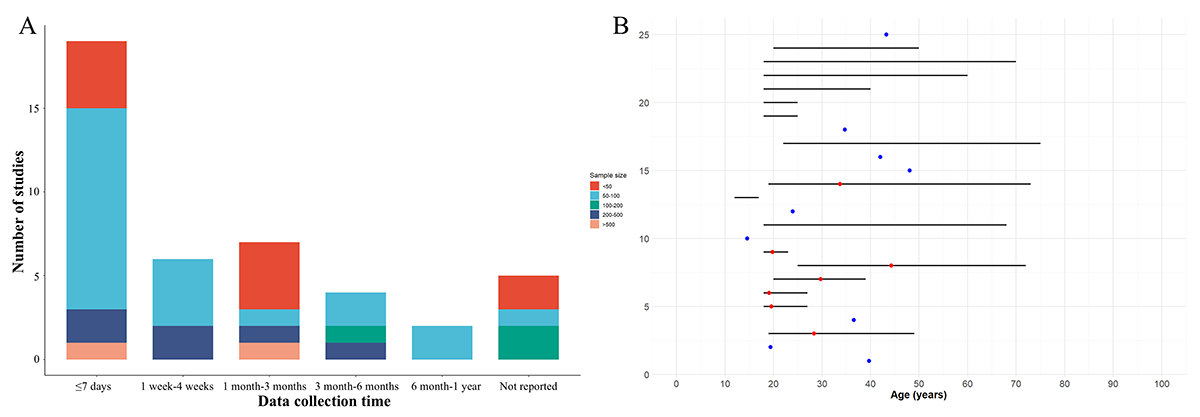
Overview of study sample characteristics and data collection duration—(A) distribution of sample size and data collection duration; (B) distribution of participant age characteristics.

Notably, as many as 76% (32/42) of the studies had sample sizes of <100 participants, with 24% (10/42) of the studies having sample sizes of <50 participants. In addition, 45% (19/42) of the studies collected passive data for <7 days, and 12% (5/42) of the studies did not report the specific duration of data collection. The distribution of sample sizes and data collection durations is shown in Figure 3A. Furthermore, the distribution of participant age characteristics can be found in Figure 3B.

Regarding the availability of datasets, 38% (16/42) of the studies did not provide explicit information, whereas the remaining studies included 31% (13/42) that provided open access datasets. A detailed list of the characteristics of the included studies is shown in Table 1, and the complete list of studies can be found in Multimedia Appendix 4 [14,15,17-19,24,25,44-79].

In addition, we conducted a detailed analysis of the funding sources for the included studies. The results revealed that 62% (26/42) of the included studies received funding, with these studies having a total of 69 funding instances. The breakdown of these funding instances showed that government agencies were the most prominent source, accounting for 57% (39/69) of instances. This was followed by funding from universities and research institutions (19%, 13/69) and nonprofit organizations or foundations (15%, 10/69). Funding from international organizations (6%, 4/69), private individuals (3%, 2/69), and corporations (1%, 1/69) was less common. This distribution indicates a high level of public sector interest and suggests that the field has relatively low appeal for industry and private capital.

**Figure 4:**
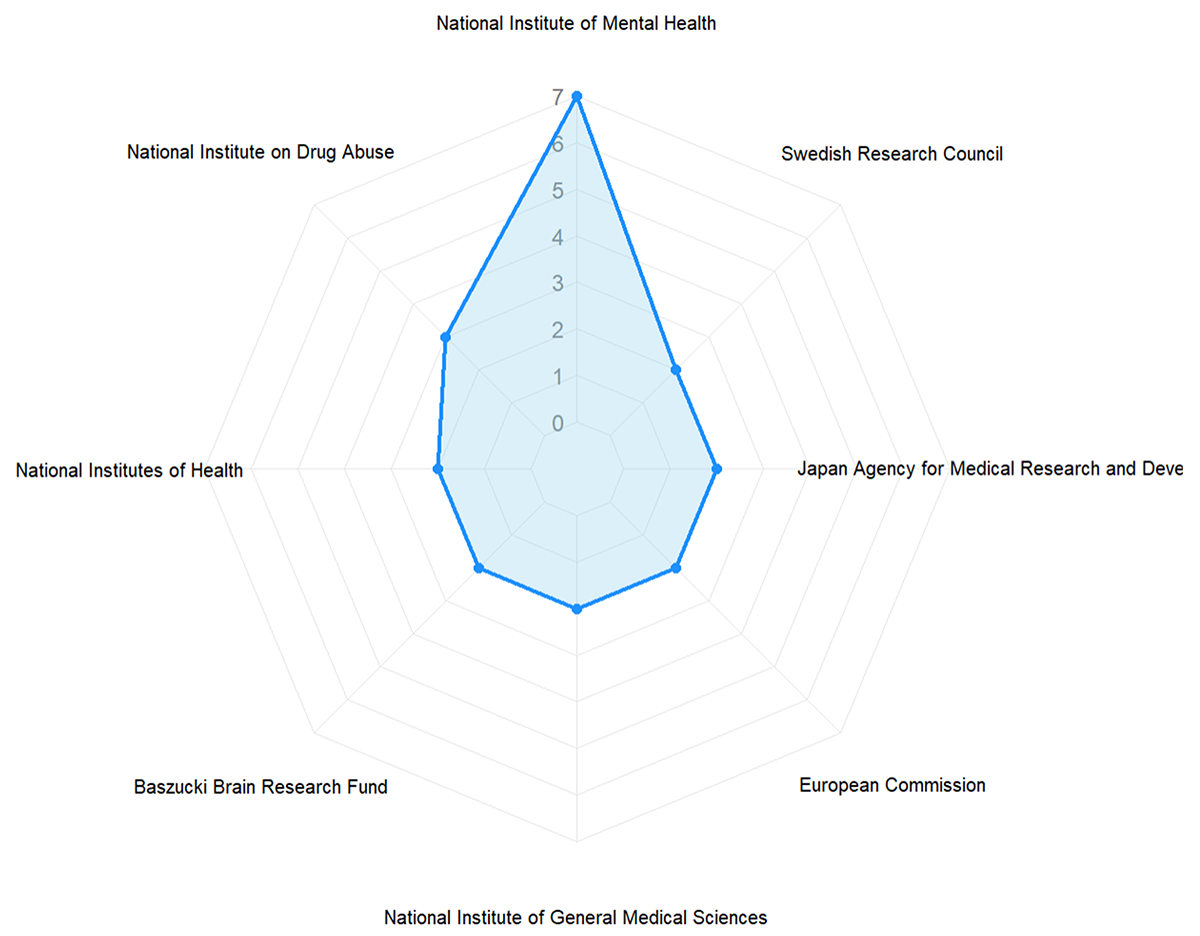
Distribution of funding frequency from major funding organizations.

Furthermore, as shown in Figure 4, the major funding agencies were the National Institute of Mental Health in the United States, which provided 10% (7/69) of the funding instances, followed by the National Institute on Drug Abuse, which contributed 4% (3/69) of the funding instances. These data not only highlight the concentration of funding sources but also provide a strong basis for further exploration of research trends and policy support in this field.

**Table 1:** Characteristics of the included studies (N=42).

Category and subcategory			Articles, n (%)		References
**Research design**					
	Cohort study	23 (55)		[15,17,18,44-62,80]	
	Cross-sectional study	14 (33)		[24,25,63-74]	
	RCT^a^	1 (2)		[75]	
	Case-control study	4 (10)		[76-79]	
**Disease categories**					
	Depression	24 (57)		[15,17,46,47,49,51-53,55-57,59-61,63,67,68,71,73,77-79]	
	Schizophrenia	6 (14)		[58,63,68,74-76]	
	Bipolar disorder	4 (10)		[19,44,48,69]	
	Anxiety	9 (21)		[14,18,24,45,47,50,65,66,70,72]	
	PTSD^b^	1 (2)		[54]	
	Attention-deficit/hyperactivity disorder	1 (2)		[25]	
	Suicidal ideation	1 (2)		[62]	
**Data collection duration**					
	6 mo-1 y	2 (5)		[19,75]	
	3-6 mo	4 (10)		[46,52,58,62]	
	1-3 mo	7 (17)		[17,48,51,53,55,61,65,70]	
	1-4 wk	5 (12)		[14,45,63,68,76]	
	≤7 d	19 (45)		[18,24,25,44,47,49,50,54,57,60,66,67,69-71,74,77-79]	
	Not reported	5 (12)		[14,15,56,59,72,73]	
**Accessibility of data**					
	Open access	13 (31)		[14,24,25,47,60,63,66,67,70-72,76,77]	
	Not open access	9 (21)		[15,44-46,51,53,54,57,62,74,78,79]	
	Not specified	16 (38)		[17-19,48-50,52,56,58,59,61,65,68,69,73,75]	
**Device types**					
	Mobile phone	20 (48)		[14,15,17,18,24,45,46,48,49,51,53,59,61,62,65,70,73-75]	
	Watch, wristband, or wrist strap	14 (33)		[19,44,46,52-55,57,60,61,65,69,74,79]	
	Research-grade accelerator	11 (26)		[25,47,50,56,63,66-68,71,76,77]	
	Others	5 (12)		[24,25,70,72,78]	

^a^RCT: randomized controlled trial.

^b^PTSD: posttraumatic stress disorder.

### Data Acquisition

#### Measurement Environment and Location

Regarding the data collection environment, 17% (7/42) of the studies conducted data collection in controlled environments, whereas 83% (35/42) of the studies were carried out in natural settings. Most studies (31/42, 74%) used pre-established measurement protocols, requiring participants to collect data during specified tasks or activities. This approach helps reduce variability between individuals and the environment, enhancing the standardization of data and providing more stable and reliable inputs for subsequent ML-based signal analysis [45,46,48,65]. In contrast, a few studies (10/42, 24%) did not implement strict collection protocols and aimed to capture signal variations in participants’ daily real-world states [24,25,47,55,67,71-73,76,77]. Although this method may introduce noise, it more accurately reflects the real psychological dynamics, providing valuable data support for applications in real-world settings.

Regarding device placement, the use of wearable devices was mostly concentrated at the wrist, with 57% (24/42) of the studies specifically fixing the device at this location. This choice is likely driven by the practical considerations of ease of long-term wear and the efficient capture of physiological signals (such as heart rate and activity levels) [81,82]. Only 5% (2/42) of the studies examined the use of wearable devices placed at 2 different locations on the body [25,53], whereas the remaining studies (40/42, 95%) opted for a single site for data collection, indicating a current reliance on single-point measurements in the research. For smartphones, most studies (18/20, 90%) did not provide specific details regarding device placement.

#### Devices and Sensors

A total of 26% (11/42) of the studies used 2 types of devices to collect passive signals, whereas the remaining 76% (32/42) used a single device. Specifically, 48% (20/42) of the studies used smartphones for data collection; 33% (14/42) used watches, wristbands, or wrist straps; 26% (11/42) used research-grade accelerometers; and 12% (5/42) used other types of devices. Among wearable devices, brands such as Fitbit, Empatica, and Actiwatch were frequently used, whereas most smartphone studies (19/20, 95%) did not specify the brand, with Android being the most commonly used operating system.

**Figure 5:**
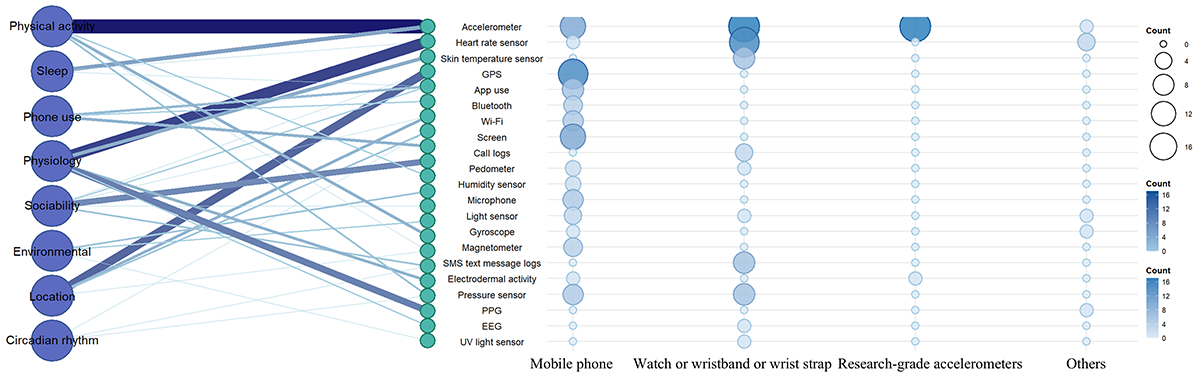
Application and distribution of different device types and sensors in passive feature signal collection for mental health assessment. Left: A network of passive data categories. Right: A bubble chart of device types and their sensors, where bubble size reflects the quantity and variety of sensors. EEG: electroencephalography; PPG: photoplethysmography.

Furthermore, we extracted the device types, sensor categories, and the passive feature types collected in each study and presented them in a network bubble diagram (Figure 5). The network diagram on the left of the figure lists the passive feature categories collected through smartphones and other sensors. For example, physical activity and physiological dimensions were particularly associated with accelerometers and heart rate sensors, highlighting the central role of these 2 sensors in monitoring these dimensions. Accelerometers were used not only to assess physical activity but also to effectively monitor sleep quality. In addition, position data collected by sensors such as GPS and Bluetooth provided valuable insights into individuals’ social behaviors and biological rhythms. The bubble diagram on the right of Figure 5 shows each device type (such as mobile phones, wearable devices, and research-grade accelerometers) and the types of sensors it is equipped with. Through this analysis, we found that smartphones offered broad sensor coverage capable of collecting data from multiple mental health dimensions simultaneously. In contrast, wearable devices such as watches or wristbands focused more on collecting physiological signals, particularly accelerometers and heart rate sensors. A detailed classification of feature categories and sensor terminology can be found in Multimedia Appendix 3.

#### Features and Behaviors

**Figure 6:**
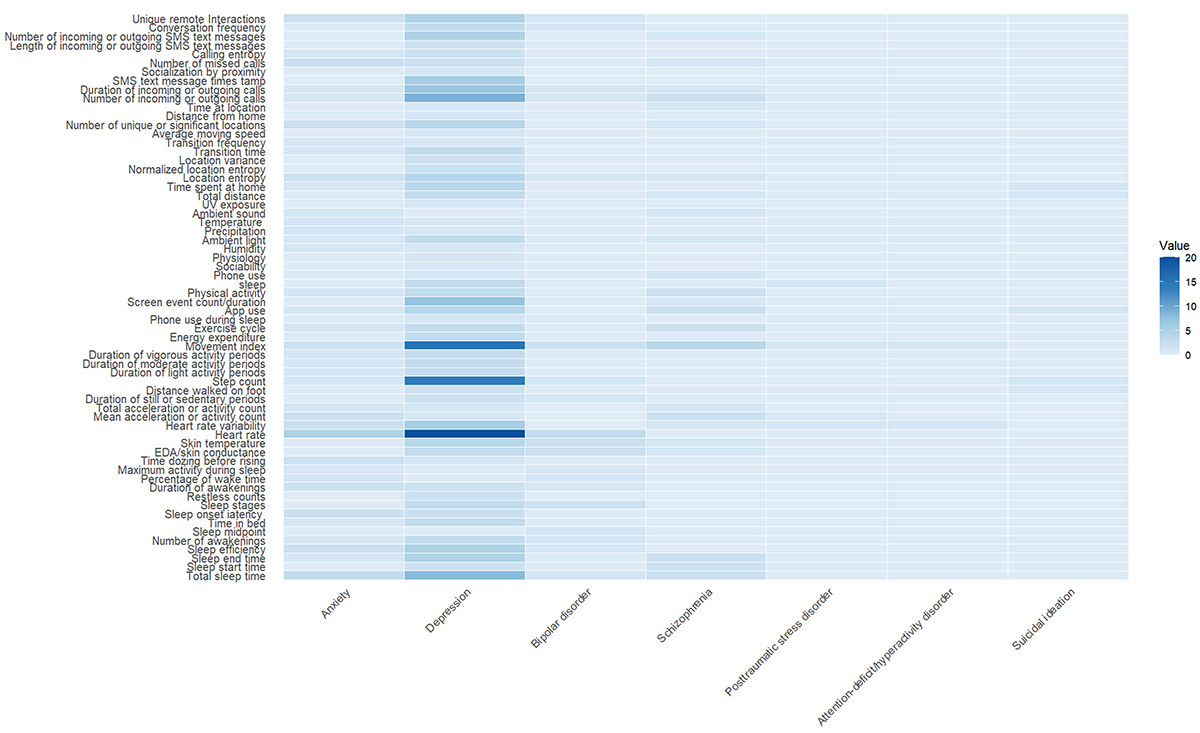
Heat map of feature use frequency across different mental illnesses. EDA: electrodermal activity.

We identified 64 passive features from wearable devices and smartphone sensors and compiled their frequency of occurrence across different mental illnesses (Figure 6). The analysis revealed that the top 5 most frequently used features were heart rate, movement index, step count, total sleep time, and the number of incoming and outgoing calls, appearing in 67% (28/42), 60% (25/42), 40% (17/42), 33% (14/42), and 31% (13/42) of the studies, respectively.

Furthermore, we categorized all features based on behavior type. The results showed that sleep-related features were the most common, with a total of 14 features. This was followed by physical activity–related features (n=11) and location-related features (n=11). The high frequency of sleep-related features indicates that sleep status was considered a crucial indicator of mental health in the existing studies. In addition, the frequent occurrence of physical activity and location information provided key data for understanding individuals’ daily rhythms and social interactions.

From the perspective of mental disorders, the frequency of feature occurrence varied across diseases. For depression, heart rate (20/24, 83% of the studies), movement index (15/24, 62%), and step count (14/24, 58%) were the most frequently used features. Anxiety disorders were primarily associated with heart rate (5/9, 56%) and total sleep time (3/9, 33%). In studies on schizophrenia (6/42, 14%), movement index (3/6, 50%) was the most common feature. Research on bipolar disorder showed a high frequency of sleep and physiological features, highlighting the significance of these aspects in the disorder. For posttraumatic stress disorder, features related to physical activity were most frequent. Attention-deficit/hyperactivity disorder was primarily associated with physical activity and physiological features. Finally, studies on individuals with suicidal tendencies (1/42, 2%) showed the highest frequency of features related to physical activity and location information.

### Data Preparation

#### Overview

The quality of passive sensor data directly impacts model performance. The included studies primarily focused on improving data quality through data preprocessing, missing data handling, and class balancing. Detailed information on data preparation methods can be found in Multimedia Appendix 4.

#### Data Preprocessing

The studies included in this review extensively applied preprocessing techniques to eliminate noise, motion artifacts, and irrelevant signal components, thereby enhancing the signal-to-noise ratio and analytical stability. A detailed breakdown of these methods by data type is provided in Table 2. Specifically, some studies (2/42, 5%) used rule-based filtering methods [44,57] to exclude abnormal physiological data (eg, heart rate exceeding a reasonable range) to prevent erroneous data from generating false correlations. For noise reduction in accelerometer, photoplethysmography, and electrocardiography (ECG) signals, researchers used low-pass or high-pass filtering [56,69,79] as well as external quality control algorithms [78]. Advanced techniques such as discrete wavelet transform were also used for both denoising and feature extraction, effectively reducing environmental interference and collection artifacts [60].

**Table 2:** Preprocessing techniques for passive sensing signals used in the included studies.

Data type	Technology	Noise or artifact type	Key parameters	Reference
Physiological	Fourth-order low-pass+second-order high-pass Butterworth filter	Baseline drift and high-frequency noise	Low-pass cutoff of 1.5 Hz, high-pass cutoff of 0.05 Hz, and extract tonic and phasic components	[69]
Physiological	Third-order band-pass Butterworth filter	Baseline drift and motion artifacts	Passband of 0.5-8 Hz, retain HR^a^-related frequencies (0.5-4 Hz corresponding to an HR of 30-240 bpm^b^)	[69]
Physiological	DRL^c^ circuit noise elimination	Baseline drift and motion artifacts	Electrode type (silver or silicone rubber) and reference electrode FpZ^d^	[70]
Physiological	Adjustable Q-factor wavelet transform	Frequency aliasing and noise interference	Q-factor=1; redundancy=3; 5-level decomposition generating 6 frequency bands	[72]
Physiological	DWT^e^	High-frequency noise and trend fluctuations	Mother wavelet (sym9, db4, or rbio3.9) and decomposition levels (1, 3, or 2)	[60]
Physiological	Resampling and normalization	High-frequency noise and dimensional differences	Target sampling rate of 500 Hz, mean and SD calculated based on the training set, and no data leakage in the test set	[78]
Physiological	*Z* score normalization	Dimensional differences and baseline drift	Mean and SD based on the training set, standardized range of μ=0, σ=1, and retain relative amplitude differences	[72]
Physiological	Signal quality index for HR variability analysis filtering	Motion artifacts and waveform distortion	Threshold=1.0; based on harmonic energy ratio	[79]
Physiological	Signal quality index for pulse rate detection filtering	Frequency-domain noise and pulse rate detection errors	Threshold=0.5; 3-min sliding window (1-min overlap)	[79]
Physiological	Band-pass filtering	Baseline drift and high-frequency or low-frequency noise	PPG^f^: 0.5-5 Hz	[79]
Physiological	Rule-based filter	Electrode detachment and circuit overload	EDA^g^ (0.05-60 μS), HR (25-250 bpm), temperature (30-40 °C), and exclude transitional segments of 5 s	[44]
Physiological	Downsampling and time alignment	Inconsistent sampling rate	Unified to 1-s time units and high-frequency signal downsampled by mean (eg, accuracy of 32 Hz → 1 Hz)	[44]
Physiological	Global normalization	Dimensional differences and uneven distribution	Scaled to (0, 1) based on minimum and maximum values calculated from the training set	[44]
Acceleration	Resampling	High-frequency motion noise and short-term fluctuations	Downsampling interval=2 h; window=48 h	[77]
Acceleration	Physically infeasible value filtering	Device malfunction and environmental interference	Exclude acceleration peaks of >10 *g*, long-term invalid values, and nonhuman motion fluctuations	[71]
Acceleration	High-pass filtering+Gaussian smoothing	Low-frequency drift and high-frequency noise	High-pass threshold of 20 mg, rolling window of 30 points (1 s), and aggregated to 1-min epoch	[56]
Acceleration	*Z* score standardization	Dimensional differences and device sensitivity noise	Calculate mean and SD using classification; formula: *Z* score = (*x* − μ)/σ	[63]
Acceleration	Third-order Butterworth filter	Device vibration and gravity interference	Cutoff frequencies: 20 Hz for high-frequency noise and 0.3 Hz for gravity separation	[17]
GPS	DBSCAN^h^ clustering algorithm	Short stop noise points and isolated location points	ε=40 m; MinPts=5	[17]
GPS	Velocity threshold filtering	Invalid positioning during motion and high-frequency sampling noise	Stationary speed≤1 km/h	[17]
GPS	Fixed-interval downsampling	Data redundancy and position fluctuations	Target sampling rate: 15 samples per hour	[17]
GPS	Spatiotemporal clustering denoising	Short-term stop and position drift	Distance=60 m (distance threshold); time=600 s (time threshold)	[45]

^a^HR: heart rate.

^b^bpm: beats per minute.

^c^DRL: driven right leg.

^d^FpZ: frontal pole zero.

^e^DWT: discrete wavelet transform.

^f^PPG: photoplethysmography.

^g^EDA: electrodermal activity.

^h^DBSCAN: density-based spatial clustering of applications with noise.

In terms of data smoothing, resampling [77,78] and framing techniques [77] were used to create stable data sequences by segmenting continuous signals into fixed windows. This approach not only smooths the data and reduces high-frequency noise but also facilitates subsequent feature extraction. In addition, 2% (1/42) of the studies reported hardware-level solutions, including noise suppression designs for electroencephalography headsets and a signal quality evaluation method that identifies and removes noise segments through statistical metrics (variance, amplitude, and kurtosis) [70].

#### Handling of Missing Data

Among the 48% (20/42) of the studies that explicitly addressed missing data, 2 primary strategies were used: data imputation and quality screening. Imputation methods were particularly diverse, including simple statistical replacements (mean [25] or median [15] imputation), ML approaches (k-nearest neighbor [KNN] [52,65] or random forest [19]), advanced filtering techniques (Kalman filter imputation [54] or median filtering interpolation [83]), and multiple imputation [14,18,49,84]. In addition, some studies (4/42, 10%) used probabilistic model–based strategies [62] that naturally integrate missing values to maintain the integrity of the dataset. This approach, compared to traditional imputation, better reflects the true structure and uncertainty of the data, thereby enhancing the robustness and predictive accuracy of the model. Another 17% (7/42) of the studies opted for deletion strategies, either directly removing low-quality segments [46,69] or merging adjacent data points [45]. Notably, only 14% (6/42) of the studies quantified and reported the proportion of missing data [14,15,19,46,52,75].

#### Data Imbalance

A total of 21% (9/42) of the studies implemented techniques to address data imbalance. The most common approach was synthetic oversampling, with 44% (4/9) of the studies using synthetic minority oversampling technique [15,60,74,76] and 22% (2/9) using adaptive synthetic sampling [47,60]. In addition, traditional upsampling [54] and downsampling [71] were applied individually. One innovative study introduced a class-weighted loss function during model training, offering a novel approach to address class imbalance that goes beyond conventional data-level techniques [55].

### Feature Engineering

#### Window Segmentation

Window segmentation plays a crucial role in passive sensor data analysis as the choice of parameters directly impacts the effectiveness of behavior and physiological pattern recognition. Among the reviewed studies, a fixed-length sliding window was the most commonly used strategy (16/42, 38%), although its implementation varied significantly in terms of window size and overlap rate. In total, 12% (5/42) of the studies systematically compared different window durations, revealing the trade-off between time resolution and noise robustness. For instance, one study tested window lengths ranging from 1 to 2048 seconds (in powers of 2) using physiological data, finding that a 32-second window achieved the best balance between accuracy and noise suppression. Short windows (eg, <32 seconds) were prone to feature fragmentation, whereas long windows (eg, >32 seconds) resulted in noise accumulation [44].

In smartphone-based social behavior analysis, a 14-day window outperformed a 7-day window as it smoothed short-term fluctuations and captured long-term trends more effectively [73]. In contrast, windows as short as 2.5 seconds were suitable for high-frequency motion signal analysis [17].

Regarding the window overlap strategy, most studies (9/42, 21%) used overlapping segmentation (with overlap rates ranging from 33% to 98%) to maximize data use and capture temporal dependencies. For example, a 3-minute window with a 1-minute overlap provided a balance between noise resistance and dynamic capture ability in free-living environments [79]. In a 50-Hz sampling rate scenario (50 data points per second), a 12-second window (600 time points) with a 90% overlap rate enabled a CNN–long short-term memory (LSTM) model to achieve an accuracy of 92.16% [72]. Furthermore, multiscale window designs (eg, 5-min windows for analyzing exercise intensity, 30-min windows for environmental interactions, and 24-h windows for circadian rhythms) were applied for hierarchical behavior modeling. By integrating features across different temporal dimensions, these designs improved the generalization ability of the models [14].

#### Feature Extraction

A total of 14% (6/42) of the studies used time-domain feature extraction methods, analyzing raw data from devices such as accelerometers, gyroscopes, and heart rate sensors to extract statistical metrics such as mean, SD, skewness, and kurtosis. These metrics were applied to assess gait [17], physical activity intensity [54,63], and sleep patterns [61], thereby providing strong support for the early diagnosis of mood disorders. Most studies (12/42, 29%) used Python as the core toolchain, implementing custom algorithms or leveraging open-source library functions (eg, NumPy and pandas) for automating the analysis of raw signals into clinical indicators. This processing workflow, standardized by design, enhanced the reproducibility of feature extraction and, through modular design, facilitated method reuse across studies.

In addition, 17% (7/42) of the studies further combined time-domain features with frequency-domain features using methods such as Fourier transform to uncover periodic fluctuations in signals, thereby facilitating deeper analysis of physiological rhythms and long-term variation patterns [14,46,56,60,66,72,79]. For example, one study used tunable Q-factor wavelet transform decomposition of ECG signals into 6 wavelet frequency bands, extracting probabilistic texture features from each band. After merging these with spatial-domain features, a highly efficient frequency domain–spatial domain feature vector was formed. These frequency-domain features effectively captured autonomic nervous system abnormalities related to anxiety, playing a critical role in achieving high model accuracy (>98.5%) [72].

Furthermore, 36% (15/42) of the studies used end-to-end learning models to automate the extraction of complex features and predict mood disorders. Autoencoder models [65,75], by constructing multilayer neural network architectures, performed nonlinear compression and reconstruction of sensor data from accelerometers and heart rate sensors, effectively extracting low-dimensional latent features to capture deep associations within behavior patterns. CNNs [24,47,77,78] excelled in modeling multichannel time-series features using 1D and 2D convolutional kernels to capture local correlations in sensor data. These models, trained end to end, directly learned the nonlinear relationships within raw data, thus avoiding the subjectivity of manual feature engineering. In addition, cross-modal fusion (eg, combining physiological signals with environmental data) further enhanced prediction accuracy, offering an efficient technical pathway for real-time monitoring using wearable devices.

#### Feature Selection

Among the included studies, 48% (20/42) used feature selection techniques. Specifically, 30% (6/20) of the studies applied filter-based methods to quickly identify key features through statistical tests. For example, one study reduced the feature set by >88% using the information gain method [77]. A total of 30% (6/20) of the studies used wrapper methods, where researchers used random forest [63], recursive feature elimination [59,63,73], and the neural network with weighted fuzzy membership function algorithm [51] to continuously evaluate the impact of different feature subsets on model performance, ultimately selecting the optimal feature combination. In addition, 40% (8/20) of the studies used embedded methods, performing automatic feature selection from hundreds of initial features through least absolute shrinkage and selection operator [58], L1 penalty [61], or L2 penalty [79], as well as intrinsic feature importance evaluations based on tree models [14,55,61] to enhance model robustness. To address the issue of ultrahigh-dimensional data, 10% (2/20) of the studies used dimensionality reduction techniques, including principal component analysis [45] and deep autoencoders [50]. For instance, one study reduced 3000 features to 200 [45], whereas another reduced 800 features to 50 [50].

#### Feature Importance Evaluation

Among the 52% (22/42) of the studies that explicitly mentioned feature importance evaluation, the methods can be broadly categorized into statistical and ML methods. Statistical methods included the use of the Cohen *d* effect size to measure the differences between abnormal behaviors before relapse and behaviors under healthy conditions, with significant features selected by setting a threshold of >0.8 [75]. Permutation importance analysis shuffles feature data and observes changes in model performance, providing a model-agnostic evaluation approach [44]. In addition, one study used standardized regression coefficients [56] and conducted ANOVA across multiple datasets to identify variables that significantly influence classification or prediction.

In terms of ML methods, various model interpretation and feature selection techniques were used to quantify the contribution of features. The Shapley additive explanations method based on game theory was used in 12% (5/42) of the studies due to its ability to intuitively quantify the marginal contribution of each feature to the model output. Some studies (2/42, 5%) combined deep neural networks [51] and multitask learning [58] to explore the role of features in complex tasks. Random forest [52,61], adaptive boosting [52], Extreme Gradient Boosting (XGBoost) [14,52,55,57,73], and the Boruta algorithm [53] quantified feature contributions by reducing node impurity or performing iterative validation. One study used feature ablation, which systematically evaluates changes in model performance based on feature subsets to identify critical feature combinations [46]. Additional methods included extra trees classifier [73], integrated gradients [65], and SVM recursive feature elimination strategies [59], which determine the most stable and crucial features through cross-validation and statistical feature selection frequency.

**Figure 7:**
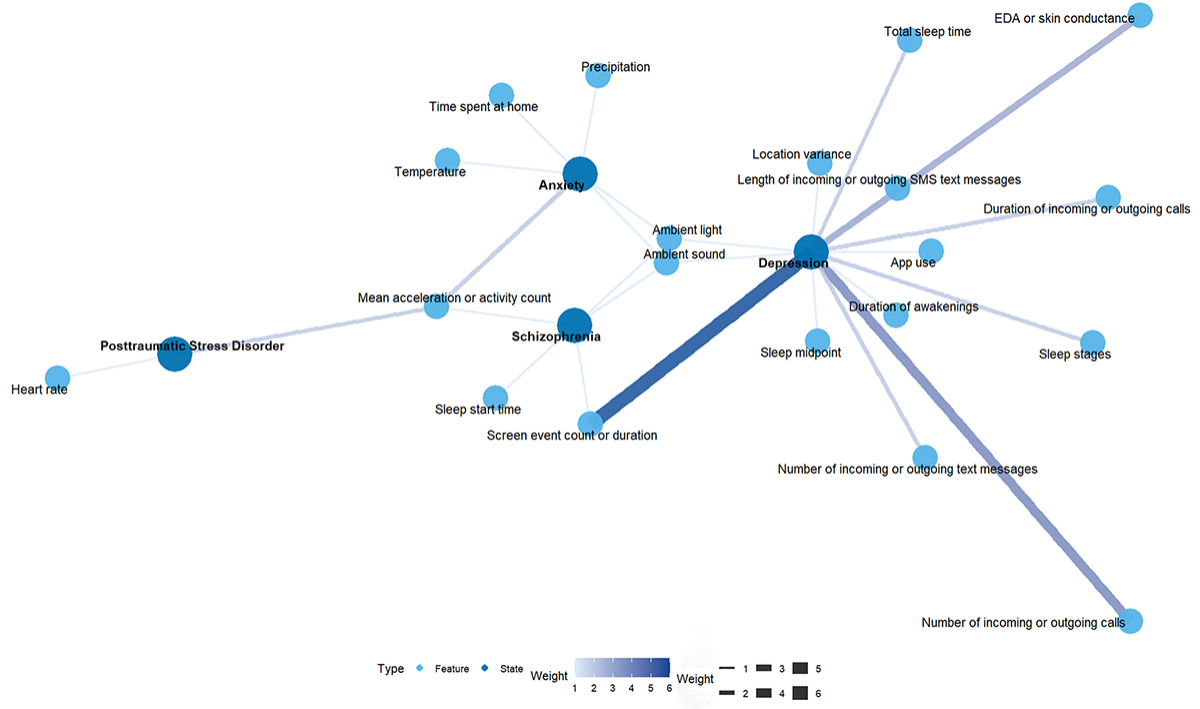
Relationship diagram of key features and mental health disorders. Feature importance was derived from the ranking or effect size across 12 related studies. Blue nodes represent features, and purple dots represent mental health disorders. EDA: electrodermal activity.

Furthermore, we reviewed 31% (13/42) of studies that explicitly ranked feature importance. Some studies (10/13, 77%) presented the ranking, whereas others (3/13, 23%) used quantitative indicators such as effect size [15,18,19,44,51-55,57,58,73,75]. To simplify the analysis and focus on the most representative features, we extracted the top 3 key features from each study and presented them in a relational diagram (Figure 7). The results indicated that, for schizophrenia, abnormalities in physical activity [75], mobile phone use behavior [58], and sleep rhythm [75] were of particular significance. For depression, changes in mobile phone use behavior [15,51-53] and social interaction [51,52,73] stood out, whereas sleep indicators [19,51,55,57] and electrodermal activity or skin conductance [44,53] also demonstrated high importance. For anxiety disorders, time at home [18] and environmental climate factors [18] were considered important features, suggesting that anxiety may be associated with reduced outdoor activity and reliance on indoor environments. For posttraumatic stress disorder, heart rate and average acceleration or activity count were identified as important indicators [54], reflecting the critical role of physiological and physical activity signals in this condition.

### ML Methods

#### Overview

**Figure 8:**
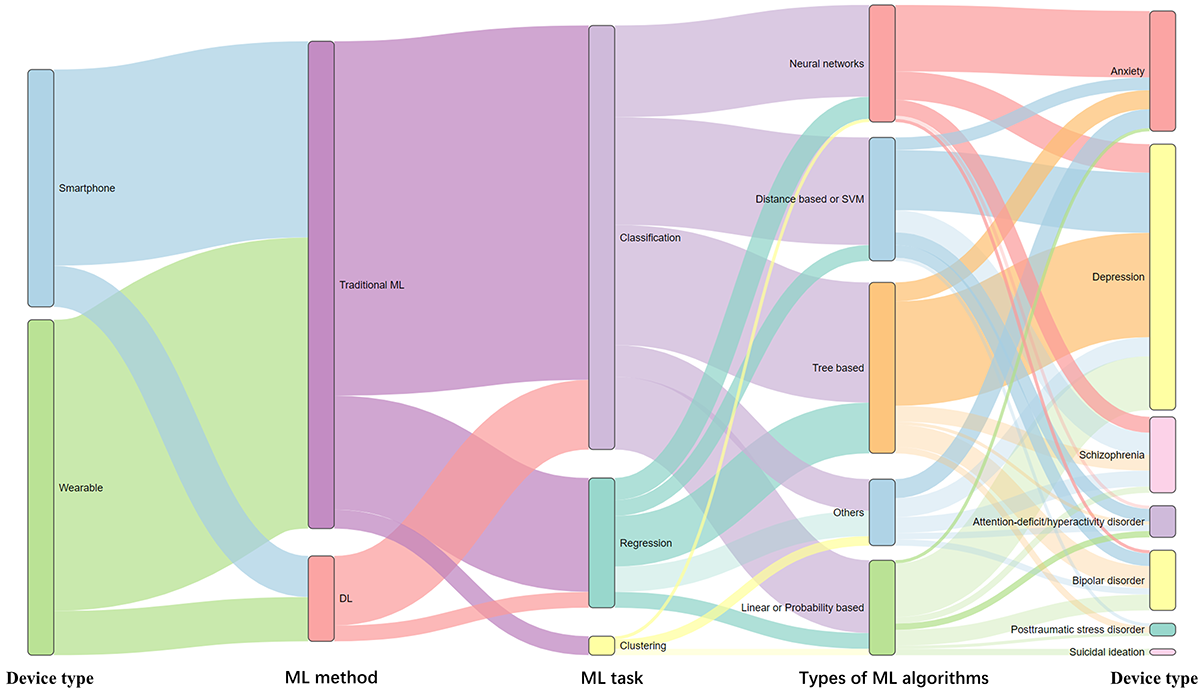
Hierarchical overview of machine learning (ML) methods used in the studies included in this scoping review. DL: deep learning; SVM: support vector machine.

In the 42 studies included, various ML algorithms were extensively used to extract behavioral features, capture abnormal patterns, and predict mental health conditions from passive, noninvasive signals collected by wearable devices and smartphones. These algorithms included both deep learning models and traditional ML methods, with many studies (12/42, 29%) achieving performance improvements through ensemble or multitask learning approaches. Figure 8 provides an overview of the application levels of these algorithms across different mental health conditions. Detailed information on the ML methods used in the included studies can be found in Multimedia Appendix 4.

#### ML Algorithms

Our analysis revealed that nearly half (19/42, 45%) of the studies used deep learning models, with deep neural networks (5/19, 26%) [24,47,51,74,76] and CNNs (5/19, 26%) [24,47,76-78] being the most commonly used architectures. These models demonstrated significant effectiveness in automatically extracting features and identifying complex patterns from high-dimensional time-series data. Notably, LSTM networks [18,71] and their variants, including bidirectional LSTM [44], hybrid CNN-LSTM [24], and ConvLSTM [24], were widely used to capture temporal dependencies, further improving the accuracy of mental health predictions. Unsupervised feature representation learning was mainly achieved through autoencoders, including fully connected neural network autoencoders [75] and deep autoencoders [65]. One study innovatively combined autoencoders with ensemble techniques such as random forest and XGBoost [65]. In addition, some studies (2/42, 5%) explored CNNs integrated with attention mechanisms [76] and hybrid architectures combining neural networks with weighted fuzzy membership functions [51].

Traditional ML algorithms played a central role, with random forest (17/42, 40%), SVM (17/42, 40%), logistic regression (11/42, 26%), and KNN (10/42, 24%) frequently used in classification tasks. Boosting algorithms, including adaptive boosting (4/42, 10%), XGBoost (13/42, 31%), and CatBoost [60], demonstrated superior performance in both regression and classification tasks. Some studies (13/42, 31%) also used linear models such as least absolute shrinkage and selection operator and Elastic Net [52], as well as decision tree [48,61,65,67,69,74], naïve Bayes [61,67,74], and ensemble methods (eg, stacking ensemble [71] and decision integration strategies [46,50,62]), to further explore data relationships and feature importance. Furthermore, spatiotemporal clustering [45] and change point detection [62] methods proved effective for extracting semantically meaningful locations from GPS data and detecting sudden behavior changes.

In terms of algorithm performance comparison, 60% (25/42) of the studies provided valuable results. Among them, 68% (17/25) focused on comparing traditional algorithms. The findings indicated that random forest demonstrated consistent performance advantages in several studies, with 12% (3/25) of the studies independently selecting random forest as the best model [52,61,67]. The average accuracy of these studies reached 0.835, and the average area under the curve (AUC) was 0.634. XGBoost also performed excellently, leading in 12% (3/25) of the studies [54,57,73], with an average AUC of 0.727 [46,48]. Methods that integrated multiple models showed unique advantages in 24% (6/25) of the studies, achieving an average accuracy of 0.816 and an average AUC of 0.701.

In comparisons between deep learning and traditional algorithms, 82% (9/11) of the studies reported that deep learning methods significantly outperformed traditional methods, with accuracy ranging from 0.85 to 0.963. Common baseline models included random forest, SVM, logistic regression, and KNN. Among these, the CNN-LSTM model demonstrated exceptional performance in one study, with an accuracy of 0.9216 in predicting anxiety disorder [72]. The 2D CNN model showed better balance across metrics, with a sensitivity of 0.75, specificity of 0.77, accuracy of 0.7672, and AUC of 0.76 [47]. Although the LSTM model exhibited a high sensitivity of 0.84, its practical application may be limited by other performance factors [71]. In total, 18% (2/11) of the studies focusing on deep learning architectures further confirmed the technical advantages of CNN-based feature extraction models (CNN-LSTM and 2D CNN) [24,47].

Table 3 summarizes the performance of each model across 4 metrics—sensitivity, specificity, accuracy, and AUC—in the included studies (note: for studies reporting multiple performance metrics for the same model, only the highest value is presented). Table 4 presents the performance data of the models selected as the best by the original studies, specifically including those that reported the 4 aforementioned metrics.

**Table 3:** Performance comparison of machine learning models across the included studies.

Model type	Sensitivity	Specificity	Accuracy	AUC^a^	Average performance
DNN^b^	0.61 [47]	0.83 [47]	0.72 [47], 0.77 [51], 0.912 [74], and 0.7029 [24]	0.73 [47]	Sensitivity: 0.61; specificity: 0.83; accuracy: 0.776; AUC: 0.73
CNN^c^	—^d^	—	0.8072 [77], 0.77 [78], and 0.7932 [24]	—	Accuracy: 0.79
AlexNet	0.45 [71]	0.82 [71]	—	0.68 [71]	Sensitivity: 0.45; specificity: 0.83; AUC: 0.68
LSTM^e^	0.84 [71]	0.26 [71]	0.8126 [77] and 0.6713 [24]	0.55 [71]	Sensitivity: 0.84; specificity: 0.26; accuracy: 0.742; AUC: 0.55
2D CNN	0.75 [47]	0.77 [47]	0.7672 [47]	0.76 [47]	Sensitivity: 0.75; specificity: 0.77; accuracy: 0.7672; AUC: 0.76
CNN-LSTM^f^	—	—	0.9216 [24]	—	Accuracy: 0.9216
Fully connected neural network autoencoder^g^	0.25 [75]	0.88 [75]	—	—	Sensitivity: 0.25; specificity: 0.88
GRU Seq2Seq^h^	0.29 [75]	0.86 [75]	—	—	Sensitivity: 0.29; specificity: 0.86
BiLSTM^i^	—	—	0.7 [44]	—	Accuracy: 0.7
Autoencoder^j^	—	—	0.87 [65]	—	Accuracy: 0.87
MLP^k^	—	—	0.81 [45] and 0.7692 [70]	—	Accuracy: 0.79
ConvLSTM^l^	—	—	0.6982 [72]	—	Accuracy: 0.698
Decision tree	0.9662 [67]	0.9737 [67]	0.9697 [67], 0.8056 [61], 0.798 [69], and 0.689 [74]	—	Sensitivity: 0.9662; specificity: 0.9737; accuracy: 0.816
Logistic regression	0.777 [67], 0.39 [71], 0.873 [79], and 0.682 [60]	0.8 [67], 0.74 [71], 0.84 [79], and 0.594 [60]	0.791 [46], 0.7885 [67], 0.859 [79], 0.64 [60], 0.7076 [70], and 0.7591 [61]	0.55 [71], 0.62 [54], 0.93 [79], and 0.638 [60]	Sensitivity: 0.68; specificity: 0.744; accuracy: 0.758; AUC: 0.685
Gradient boosting	—	—	0.791 [46] and 0.798 [72]	—	Accuracy: 0.795
Naïve Bayes	0.6418 [67]	0.9078 [67]	0.77516 [67], 0.8056 [61], and 0.704 [74]	—	Sensitivity: 0.6418; specificity: 0.9078; accuracy: 0.762
Random forest	0.9989 [67] and 0.673 [60]	0.95 [67] and 0.602 [60]	0.9865 [67], 0.9824 [63], 0.64 [60], 0.8769 [70], 0.8542 [61], 0.719 [74], and 0.7837 [72]	0.63 [54] and 0.638 [60]	Sensitivity: 0.836; specificity: 0.776; accuracy: 0.835; AUC: 0.634
XGBoost^m^	0.7 [66], 0.9662 [67], 0.73 [57], and 0.648 [60]	0.955 [66], 0.9931 [67], 0.81 [57], 0.82 [73], and 0.561 [60]	0.82 [15], 0.953 [67], 0.76 [57], and 0.607 [60]	0.892 [66], 0.7 [54], 0.712 [55], and 0.605 [60]	Sensitivity: 0.761; specificity: 0.828; accuracy: 0.816; AUC: 0.727
SVM^n^	0.9391 [67], 0.63 [60], and 0.843 [17]	0.897 [17], 0.9736 [67], 0.79 [59], 0.75 [73], and 0.571 [60]	0.75 [51], 0.872 [17], 0.8624 [67], 0.602 [60], 0.7786 [61], and 0.79 [74]	0.61 [54] and 0.601 [60]	Sensitivity: 0.804; specificity: 0.796; accuracy: 0.776; AUC: 0.606
ANN^o^	0.767 [17]	0.915 [17]	0.854 [17]	—	Sensitivity: 0.767; specificity: 0.915; accuracy: 0.854
CatBoost	0.687 [60]	0.588 [60]	0.64 [60]	0.58 [60]	Sensitivity: 0.687; specificity: 0.988; accuracy: 0.64; AUC: 0.58
KNN^p^	0.695 [17], 0.99 [67], and 0.61 [60]	0.846 [17], 0.9736 [67], and 0.55 [60]	0.825 [17], 0.9865 [67], 0.582 [60], 0.8267 [61], and 0.752 [74]	0.58 [60]	Sensitivity: 0.765; specificity: 0.79; accuracy: 0.794; AUC: 0.58
Ensemble model	0.846 [50], 0.712 [19], 0.95 [76], 0.74 [71], and 0.682 [60]	0.527 [50], 0.856 [19], 0.43 [71], and 0.594 [60]	0.881 [46], 0.687 [50], 0.851 [77], 0.801 [19], 0.64 [60], 0.9995 [72], 0.9159 [69], and 0.755 [74]	0.695 [50], 0.86 [19], 0.61 [71], and 0.638 [60]	Sensitivity: 0.786; specificity: 0.602; accuracy: 0.816; AUC: 0.701

^a^AUC: area under the receiver operating characteristic curve.

^b^DNN: deep neural network.

^c^CNN: convolutional neural network.

^d^Not available.

^e^LSTM: long short-term memory.

^f^CNN-LSTM: a hybrid model combining CNN and LSTM.

^g^An autoencoder composed of fully connected layers used for nonlinear dimensionality reduction or feature learning.

^h^GRU Seq2Seq: gated recurrent unit sequence-to-sequence model.

^i^BiLSTM: bidirectional LSTM.

^j^An unsupervised learning model.

^k^MLP: multilayer perceptron.

^l^ConvLSTM: an LSTM integrated with convolutional operations, suitable for spatiotemporal sequence prediction.

^m^XGBoost: Extreme Gradient Boosting.

^n^SVM: support vector machine.

^o^ANN: artificial neural network.

^p^KNN: k-nearest neighbor.

**Table 4:** Best reported performance of the models in the included studies.

Model type	Sensitivity	Specificity	Accuracy	Area under the ROC^a^ curve
Fully connected neural network autoencoder^b^	0.25 [75]	0.88 [75]	—^c^	—
Autoencoder^d^	—	—	0.87 [65]	—
CNN-LSTM^e^	—	—	0.9216 [72]	—
Random forest	0.9989 [67]	0.95 [67]	0.9865 [67], 0.8769 [70], and 0.8542 [61]	—
XGBoost^f^	0.73 [57]	0.81 [57] and 0.82 [73]	0.76 [57]	0.7 [54]
AlexNet	0.45 [71]	0.82 [71]	—	0.68 [71]
CatBoost	0.687 [60]	0.588 [60]	0.64 [60]	0.58 [60]
SVM^g^	0.843 [17]	0.897 [17]	0.872 [17]	—
DNN^h^	—	—	0.77 [51] and 0.912 [74]	—
Ensemble model	0.846 [50] and 0.712 [19]	0.527 [50] and 0.856 [19]	0.881 [46], 0.687 [50], 0.851 [77], 0.801 [19], 0.9995 [72], and 0.9159 [69]	0.696 [50] and 0.860 [19]

^a^ROC: receiver operating characteristic curve.

^b^An autoencoder composed of fully connected layers used for nonlinear dimensionality reduction or feature learning.

^c^Not available.

^d^An unsupervised learning model.

^e^CNN-LSTM: a hybrid model combining convolutional neural network and long short-term memory.

^f^XGBoost: Extreme Gradient Boosting.

^g^SVM: support vector machine.

^h^DNN: deep neural network.

#### Validation Methods and Performance Metrics

K-fold cross-validation was the primary validation strategy (19/42, 45% of the studies), with 5-fold (5/19, 26%) and 10-fold (11/19, 58%) cross-validation being the most commonly used. Leave-one-out cross-validation (9/42, 21%) was also frequently mentioned. In addition, repeated stratified 10-fold cross-validation [55] and sliding window cross-validation [18,49] were applied to time-series data in studies focused on dynamic prediction. A total of 12% (5/42) of the studies used multiple validation methods [15,45,52,58,67,78], and 5% (2/42) of the studies used 4 different cross-validation schemes [52,58]. Notably, only 2% (1/42) of the studies conducted external validation [56].

Performance evaluation used task-specific metrics. Classification studies primarily reported accuracy, sensitivity, specificity, precision, recall, *F*_1_-score, and AUC. One study introduced the true positive rate–to–false positive rate ratio to optimize the sensitivity-specificity trade-off [75]. In regression analysis, metrics such as mean squared error, root-mean-squared error, mean absolute error, mean absolute percentage error, *R*^2^, and the Pearson correlation coefficient (eg, *r*=0.587) were used. These were supplemented with the Brier score [66], Matthews correlation coefficient [63,79], κ statistic [70], and Bland-Altman plots [56] to assess the robustness of the results.

## Discussion

### Methodological Challenges and Data Limitations

For a long time, mental health monitoring has relied on subjective assessment methods such as clinical interviews and self-report scales, which are often limited by ecological validity and recall bias [4,5]. With the widespread use of smartphones and wearable devices, passive sensing technologies now enable continuous, noninvasive collection of multidimensional data on user activity, heart rate, sleep, social interactions, and more in natural environments. This provides more objective, granular, and quantitative information on mental health status [14]. However, the multisource, time-series data generated by these sensors often exhibit characteristics such as high dimensionality, redundancy, and noise, making it difficult for traditional statistical methods to effectively capture the associations between underlying behavioral patterns and psychological indicators [20].

ML, particularly deep learning models, offers advantages in automatic feature extraction and nonlinear pattern learning. These models can uncover subtle emotional and cognitive features from complex sensor data, significantly improving the accuracy of mental state detection and prediction [24]. While several reviews have focused on passive sensing technologies themselves or provided single-technology summaries for specific mental disorders, there is a lack of a comprehensive review that systematically evaluates the combination of passive sensing and ML for mental health monitoring, including the entire technical framework and practical outcomes.

This study was conducted in this context and, for the first time, comprehensively reviewed and integrated research on the combination of passive sensing and ML for mental health monitoring from 2015 to 2025. It covered key aspects such as data acquisition, preprocessing, feature engineering, algorithm selection, and validation. The aim was to reveal the relationships between digital biomarkers and various mental disorders; assess the strengths and weaknesses of different ML models; and summarize existing challenges regarding sample size, data quality, model generalization, and ethical compliance. The following discussion will focus on methodological limitations, technical optimization pathways, clinical validation requirements, and ethical considerations.

Analysis revealed that cohort studies dominate the field (23/42, 55%), reflecting a consensus on the importance of longitudinal data collection. However, the sample sizes were generally small, with a median of only 60.5 (IQR 54-99) participants. Furthermore, 76% (32/42) of the studies included <100 participants, with 24% (10/42) of the studies having <50. This small sample size increases the risk of model overfitting, which undermines the robustness of the conclusions, especially in the validation of complex models such as bidirectional LSTM [44]. In addition, nearly half (19/42, 45%) of the studies had data collection periods of <7 days, whereas the pathological features of mental disorders typically require extended observation periods (eg, manic episodes in bipolar disorder often last several weeks [85]). Short-term monitoring may struggle to effectively distinguish transient mood fluctuations from true disease states.

Regarding data sharing, only 31% (13/42) of the studies provided open access data, and 38% (16/42) of the studies did not clearly specify data availability, which, coupled with a lack of standardized assessment protocols in the field, hinders result reproducibility [28]. It is noteworthy that only 2% (1/42) of the studies conducted external validation. The widespread absence of external validation, which is crucial for assessing model generalizability and avoiding overestimation of performance due to sample bias or overfitting, further highlights the limitations in model dissemination and validation in the current research [86].

The bottlenecks related to insufficient dataset size and lack of diversity are primarily constrained by the sensitivity of mental health data, the complexity of cross-study collaborations, and the challenges of long-term monitoring. The sensitivity of mental health data necessitates strict privacy protection requirements, significantly limiting the availability of data [87]. Moreover, conducting cross-center, cross-cultural studies requires coordinating multiple institutions and facing issues such as inconsistent technical standards and high data heterogeneity, which further increase collaboration costs [88]. More critically, the pathological features of mental disorders often require long-term monitoring for accurate capture, yet patient compliance, device battery life, and the continuous investment of research resources present practical barriers [89]. In response to these challenges, recent research has explored solutions from both system architecture and algorithm optimization perspectives.

At the system level, scalable digital data collection platforms (eg, Intelligent Sensing to Inform and Learn [90] and AwarNS [91]) have reduced the implementation barriers for multicenter studies through modular design and privacy protection mechanisms. Intelligent Sensing to Inform and Learn provides standardized sensor toolkits for iOS and Android devices, whereas AwarNS integrates a *sense-analyze-act* framework, supporting the closed-loop process from data collection to intervention and providing infrastructure for large-scale heterogeneous data collection.

At the algorithm level, researchers have reduced reliance on labeled data through unsupervised learning [92] and used data augmentation and generative adversarial networks to synthesize virtual samples, thereby expanding training datasets [93]. In addition, federated learning has enabled cross-institution data collaboration through distributed training, improving model generalization capabilities while maintaining privacy protection [94]. Although these methods have not fully resolved the data scarcity issue, they offer feasible paths to balance data scale and quality.

### Technological Implementation and Multimodal Integration

In terms of technological implementation, 76% (32/42) of the studies used a single device. While this reduces implementation complexity, it may overlook other potentially important signal dimensions. In addition, most studies (24/42, 57%) opted for wrist-worn devices, which, while convenient, have limitations in fully monitoring physiological and behavioral signals. For example, head-worn devices can capture electroencephalography activity [70], whereas chest sensors are better suited for monitoring cardiopulmonary function [25]. Future research should consider the integration of multilocation devices (eg, head, chest, and waist) to enhance the multidimensionality and consistency of the data, as well as explore multimodal fusion architectures (eg, CNN-LSTM combining physiological and GPS data [24]).

The quality of passive sensing data faces dual challenges in device compliance and technological reliability. Studies have shown that individual differences in device use compliance—such as irregular wear, forgotten charging, or voluntary discontinuation—often result in significant gaps and noise in the data. For instance, Mullick et al [52] reported a 67.74% data loss rate in their adolescent sample due to compliance issues such as forgetting to wear the device, charging requirements, and skin irritation. This issue may be further exacerbated by variations in user motivation. The intrusiveness of long-term monitoring may lead to “device fatigue” [95,96], whereas socioeconomic factors such as the availability of charging infrastructure, as well as privacy concerns across different cultural backgrounds, can also affect users’ willingness to continue participation.

From a technological perspective, consumer-grade wearable devices (eg, Fitbit) often use closed algorithms that may obscure the true quality of the data [55], and device malfunctions can exacerbate data sparsity [18,44,47,65]. To address these challenges, future research could focus on 3 main areas: the development of adaptive reminder systems based on individual use patterns, the establishment of effective gamification incentives, and the optimization of lightweight device designs (eg, smart rings) [97-99]. When combined with intermittent active feedback methods such as ecological momentary assessment [100], these solutions may help establish a more robust multimodal data validation system.

Regarding data preprocessing, noise reduction techniques such as outlier detection based on statistical features and time-domain filtering are effective at reducing interference. However, due to the differing signal characteristics of various sensors (eg, accelerometers, photoplethysmography, and ECG), there remains a lack of standardized practices in the field [101]. More importantly, few studies have systematically evaluated the actual impact of these preprocessing methods on the final model performance, resulting in a lack of empirical evidence to guide method selection.

The handling of missing data also presents challenges. Traditional methods such as mean imputation or KNN imputation can partially restore data completeness, but they may distort the true characteristics of physiological signals. On the other hand, directly deleting low-quality data may lead to the loss of critical clinical information [102]. Recent studies on motor activity data in schizophrenia have proposed a day-night difference-sensitive heuristic imputation method that preserves the key biological marker of nocturnal activity suppression while achieving a diagnostic accuracy of 94% [76]. This provides a reference framework for preprocessing similar data types.

In addition, techniques such as synthetic oversampling offer potential solutions for addressing class imbalance. However, the synthetic samples generated often fail to adequately reflect the complex temporal patterns in real data, leading to potential model overfitting or poor performance in practical applications [103,104]. A deeper challenge lies in the fact that many studies [15,19,45,46,48,51,57,73] overlook the details of time granularity, simplifying sensor data into daily summaries. This may miss micropatterns that signal symptom changes. For example, sleep fragmentation features in patients with bipolar disorder may be entirely masked in daily average sleep duration metrics [19].

The selection of window length, overlap rate, and feature selection strategies significantly impacts the performance and generalization ability of the model. The studies included in this review indicate that the window size in time-series analysis ranges from a few seconds to several days, and its choice directly affects signal integrity and model discriminability. For example, in high-frequency motion data analysis, a 2.5-second window effectively captures fine-grained behavior fluctuations such as walking and resting (with step count accuracy reaching 99.2%). However, it is also prone to interference from motion artifacts and other noise, requiring the application of a third-order Butterworth filter to enhance signal quality [17]. In contrast, a 48-hour window in circadian rhythm analysis can effectively capture periodic movement patterns. However, a long window may obscure short-term behavioral abnormalities in patients with depression—such as sudden decreases in activity or disruptions in movement patterns, which could be smoothed out by the 48-hour window, leading to the model missing transient features related to fluctuations in the patients’ condition [77]. This suggests that there is an *optimal threshold* for window length—shorter windows may capture fine-grained behavioral fluctuations but may amplify noise, whereas longer windows may mask transient psychological state changes, requiring dynamic adjustments based on the target behavior’s timescale.

The range of overlap rates also plays a critical role in model performance, balancing sample independence and data use efficiency. Khan et al [24] systematically analyzed the impact of overlap rates (0%-90%) on time-series model performance in a study on anxiety-related behaviors. The results indicate that a high overlap rate increases sample density through resampling, improving the model’s ability to capture brief behaviors, but it also introduces data redundancy, significantly increasing the risk of overfitting. A low overlap rate reduces sample correlation but may miss key state transitions. This research provides empirical evidence for the dynamic optimization of overlap rates in time-series analysis, highlighting the need to consider the target behavior’s duration and the model’s generalization requirements when adjusting parameters.

Differences in feature selection methods directly influence model complexity and interpretability. Variations in feature space search strategies, previous assumptions, and computational costs across different techniques can lead to significant divergence in the key variables captured by the model and their clinical relevance. For instance, brute force search, which exhaustively tests feature combinations (generating 14,892 models), can cover more potential patterns but may introduce redundant features, leading to overfitting and reduced interpretability [56]. In contrast, stepwise ML algorithms based on biological priors (such as circadian rhythm theory) progressively select features. While this sacrifices some model diversity, it allows the focus to remain on core variables with clinical mechanisms, and the resulting feature interpretability is stronger [15]. In cross-modal scenarios, embedded methods (eg, Depress-Deep Convolutional Neuro-Fuzzy combining CNN with statistical features) enhance accuracy to 85.1% by removing 88% of redundant features, whereas traditional SVM recursive feature elimination methods, reliant on manual feature engineering, perform more limitedly when multimodal data are missing [77]. Therefore, the choice of method should balance data-driven pattern discovery with theory-driven mechanism interpretation, optimizing based on the research goals (exploratory or confirmatory) and data characteristics (such as modality and completeness).

### Associations Between Passive Features and Mental Disorders

The included studies suggest underlying patterns linking passive digital features to mental disorders; however, these findings are predominantly exploratory and require further validation. Notably, the significant methodological heterogeneity across studies—in terms of study quality, data acquisition methods, and reporting standards—limits the interpretability of pooled associations between digital features and affective states. Nevertheless, we would be remiss in ignoring the growing consensus on the relationship between digital features and emotional states. Consequently, we provide a synthesis of these preliminary findings, emphasize their preliminary nature, and urge readers to consider these limitations when interpreting the results.

In depression-related research, heart rate (20/42, 48%), movement index (15/42, 36%), and step count (14/42, 33%) were the most frequently reported features; these metrics correlate strongly with core depressive symptoms such as psychomotor retardation associated with anhedonia [105]. However, although smartphone use behaviors ranked highly in feature importance analyses, only 17% (7/42) of the studies actually collected these data, likely reflecting data acquisition challenges—phone call records raise privacy concerns, whereas step counts can be easily extracted from commercial wearables [19]. In anxiety disorder research, physiological and sleep characteristics such as heart rate (5/42, 12%) and sleep duration (3/42, 7%) were commonly observed. However, the importance of environmental and behavioral factors such as time spent at home suggests that future studies should focus on enhancing the integration of environmental sensors. In schizophrenia research, the movement index (3/42, 7%) was predominant, which may be associated with motor impairments caused by antipsychotic medications [106].

### Feature Engineering and Model Selection

Window segmentation lays the foundation for constructing stable data sequences by structuring inputs for feature extraction and, via overlapping windows, increasing the number of training samples to enhance model generalizability. However, there were variations in the selection of window sizes across the studies, and no unified guidelines have been established. In feature extraction, deep learning–based approaches have emerged as the dominant strategy because they automatically capture complex feature interactions and markedly improve predictive performance. CNNs in particular excel at automated feature extraction but rely on large-scale training datasets, increasing the risk of overfitting [107]. To mitigate this issue, previous studies have suggested that layerwise dropout regularization is an effective strategy, although the generalizability of such architecture optimization solutions across different datasets still requires further validation [47].

It is important to note that, although end-to-end deep models offer performance advantages, their *black box* nature limits their interpretability in clinical applications [108]. Some studies have introduced Shapley additive explanations [25,54] and integrated gradients [47] to enhance transparency, but core issues of model explainability persist. In some research, efforts to improve interpretability have led to oversimplified feature spaces (eg, retaining only 3 core features [65]). While this may facilitate clinical decision-making, it could result in the loss of multidimensional information essential for mental health monitoring. This highlights the ongoing challenge in model development to balance clinical interpretability with the need for sufficient representation of symptom heterogeneity.

In model selection, current studies retain the application of traditional ML methods (such as random forest, SVM, and logistic regression) while continuously exploring the potential of deep learning models. CNNs and their variants deliver superior performance for time-series data processing, nonlinear relationship modeling, and automated feature derivation; however, their substantial computational demands hinder deployment on edge and wearable devices, further intensifying the complexity-interpretability trade-off [24,47]. Autoencoders offer an unsupervised dimensionality reduction approach that opens new avenues for feature engineering. Notably, Baygin et al [72] developed a lightweight model based on a probabilistic binary pattern, which not only achieved >98.5% classification accuracy in ECG signal–based anxiety detection but also significantly reduced computational load. Their model, with a limited but highly interpretable feature set, offers a new paradigm for balancing performance and interpretability.

Furthermore, there is mounting evidence suggesting that personalized models yield superior predictive accuracy. For example, individualized LSTM models explain up to 39% of within-subject variance in anxiety symptoms—far exceeding the explanatory power of traditional cohort-based models [18]. These findings highlight the critical importance of modeling individual differences in mental health prediction.

### Clinical Translation and Ethical Considerations

Passive sensing data often involve highly sensitive personal information. However, only 14% (6/42) of the studies explicitly mentioned anonymization procedures [44,48,51,55,59,62] despite the fact that data anonymization and encryption are fundamental requirements for ensuring privacy and security. Moreover, as research may involve repeated monitoring and prolonged data collection, informing participants and obtaining their informed consent, as well as ensuring their right to withdraw consent for data use, are crucial issues that must be addressed when translating such technologies into clinical practice.

Insufficient sample representativeness is another significant limitation. Most studies (23/42, 55%) [53] relied on single-center or specific datasets (such as university student populations [14,18,45,49] or outpatient hospital patients [15,19,46,48,51,61,62,69]), which often exhibit high homogeneity in dimensions such as age, gender, and ethnicity, making it difficult to represent a broader population. This distribution imbalance may lead to decreased model performance in other populations and even trigger systemic biases. Future research should enhance model generalizability by expanding sample diversity and using statistical correction methods (such as multiple comparison corrections). In addition, insufficient model interpretability may undermine physicians’ trust in algorithmic results, potentially affecting clinical decision-making [109].

The generalizability of current research findings is further limited by the lack of representation across cultural and socioeconomic backgrounds. Due to differences in cultural norms, stigma, and access to technology, mental health symptoms and behavioral patterns (eg, social interactions and mobility) may differ significantly across cultures [110]. For example, in some Asian cultures, the concept of *family honor* may lead individuals to reduce their use of public social applications (eg, social media engagement) due to psychological distress while increasing the use of private communication apps (eg, one-on-one messaging) [111]. In African American communities, distrust of mental health services may manifest as a preference for avoiding mental health monitoring applications that require sensitive data authorization or increased reliance on anonymous communication platforms that do not require real name registration, resulting in systematic bias in passively collected data such as location tracking and voice features [112]. In addition, socioeconomic disparities such as lower device ownership and unstable internet access in low-income or rural communities often lead to sparse or noisy data, affecting data quality [113].

In addition, current research primarily validates the correlation between passive monitoring features and mental health, but due to the lack of causal inference, it remains unclear whether these features can serve as effective intervention targets or clinical prediction biomarkers [18,45,65]. Furthermore, most studies in this review (26/42, 62%) relied on government funding, with industry involvement at only 1% (1/69), which may delay the technology translation and practical application process. Moreover, most studies (39/42, 93%) focused on the analysis of a single disease, neglecting common comorbidities. For example, the comorbidity rate between depression and anxiety is as high as 25% [114]. Therefore, future algorithm designs should aim to identify cross-diagnostic features, thereby providing a more comprehensive assessment of an individual’s mental health risks.

In terms of ethical deployment, addressing user-centered challenges is critical. Long-term adherence is often hindered by concerns about data privacy or perceived utility, especially in clinical populations. Transparent, dynamic consent interfaces and the provision of personalized feedback can effectively alleviate privacy concerns and enhance technology acceptance [115]. Furthermore, when integrating passive sensing technologies into existing health care workflows, it is essential to consider the digital literacy differences between clinicians and patients. Therefore, developing specialized training programs is crucial to ensure equitable adoption [116].

Although this study systematically reviewed the potential of passive sensing technologies in mental health monitoring, it must be acknowledged that the field currently lacks unified device approval standards, which has become a key bottleneck in the clinical translation of these technologies. Current regulatory frameworks primarily address traditional medical devices, whereas mental health monitoring technologies such as smartwatches and smartphone sensors do not yet have established uniform regulatory requirements due to their passive data collection methods, device heterogeneity, and diverse application contexts [117,118]. For example, while the US Food and Drug Administration has launched a pilot program for digital health technology precertification, specific standards for continuous mental health monitoring are still absent [119]. Similarly, the European Union’s Medical Device Regulation includes AI algorithms but does not specify how to assess the reliability of passively collected data [120]. This situation leads to potential fluctuations in model performance due to device changes, making it challenging to meet the clinical diagnostic requirements for consistency and reproducibility.

### Strengths and Limitations

To the best of our knowledge, this is the first scoping review assessing the current status and development trends of ML techniques in passive mental health monitoring based on wearable device and smartphone sensor data. Compared to previous similar studies, this review has the following advantages. First, it is the first to provide a comprehensive analysis of the entire technological process from data collection to model validation, addressing the gap in previous research that only covered partial technological aspects. Second, by integrating data from 42 empirical studies, we explored the associative patterns between different mental disorders and behavioral traits, covering a broader range of disease types and feature dimensions than most similar reviews. In addition, this study highlights the limitations of existing methods in areas such as sample representativeness, data quality, and model generalizability while proposing paths for future technological improvements, clinical validation, and ethical practice. These contributions provide a more comprehensive assessment of the current state of methodologies in the field and offer technical references and practical insights for further optimizing the application of passive sensing technologies in mental health monitoring.

However, this study also has some limitations. First, in terms of literature search, while we used a cross-disciplinary database co–search strategy, the language and literature selection criteria (only including English-language journal articles and excluding conference papers and other nonjournal literature) may have led to the omission of important research from non–English-speaking countries and time-sensitive technological solutions from conference reports. Moreover, although MeSH (Medical Subject Headings) terms were considered, a keyword-based approach was prioritized to capture emerging terminology and maintain consistency across databases; this may have introduced some noise but ensured broader coverage of interdisciplinary literature. Second, regarding the algorithm performance comparisons presented in our results, it should be noted that these quantitative findings reflect model performance under specific study conditions. Due to substantial methodological variations in experimental designs, evaluation metrics, and data characteristics across the studies, these results are more suitable for descriptive analysis of current research trends rather than definitive conclusions about algorithmic superiority. Third, our findings may be partially influenced by the broader methodological limitations of the included studies, such as small sample sizes, potential overfitting of models, unclear generalizability of results, and differences in measurement metrics and outcome definitions across studies. These factors may affect the robustness and applicability of the conclusions. Moreover, although we strictly adhered to the PRISMA-ScR guidelines, applied clear inclusion and exclusion criteria, and systematically recorded risk of bias and other relevant factors, the broad scope of health outcomes and the differences in study designs somewhat limit our ability to comprehensively assess the overall quality of the studies [121].

### Future Research Directions

Despite the significant potential of passive sensing technologies in mental health monitoring, substantial breakthroughs are still needed to translate exploratory research into clinically practical tools. On the basis of a systematic evaluation of current limitations and opportunities, we propose a comprehensive development road map aimed at addressing methodological, technical, clinical, and ethical challenges while promoting the translation of research outcomes into practice.

#### Enhancing Data Quality and Standardization

Current research is often limited by small sample sizes and short observation periods. Future studies should focus on large-scale longitudinal research with cohorts that encompass diverse population characteristics. Standardized protocols for data collection, preprocessing, and reporting should be established to ensure comparability and reproducibility across studies. In addition, extending the monitoring window is crucial for capturing the dynamic evolution of mental health states. There is also a need to develop cross-platform open databases to facilitate data sharing among researchers and provide support for algorithm validation and cross-study integration.

#### Facilitating Clinical Translation and Ethical Practices

Integrating passive sensing technologies into clinical practice requires rigorous external validation in real-world health care settings, along with the design of user interfaces that are compatible with clinical workflows. Establishing clear regulatory approval pathways and clinical application standards is essential for transitioning technology from research to application. Future research should collaborate with regulatory bodies (eg, the Food and Drug Administration and International Medical Device Regulators Forum), clinical experts, and technology developers to jointly establish approval standards for passive mental health monitoring. These standards should define device performance parameters, data quality control processes (eg, setting thresholds for missing data), and clinical validation criteria to expedite the safe and reliable integration of passive sensing technologies into real-world health care environments [122]. Implementing scientific approaches to understand clinicians’ adoption barriers and patients’ willingness to use these technologies will enhance acceptance. From an ethical perspective, privacy protection measures (such as differential privacy [123] and dynamic informed consent mechanisms [115]), bias detection, and fairness assurances must be enforced to ensure the fairness of algorithms across different genders, races, and socioeconomic groups.

#### Optimizing Algorithm Architecture and Interpretability

Future algorithm development should seek an optimal balance among model complexity, interpretability, and computational efficiency. By combining deep learning with explainable AI techniques, transparent and efficient hybrid models can be designed. In addition, solutions such as lightweight models should be explored to improve algorithm efficiency while maintaining predictive performance. The adoption of frameworks such as federated learning should prioritize the development of personalized modeling approaches to capture individual differences in symptom manifestations. Furthermore, introducing causal inference methods will help identify digital biomarkers with true clinical intervention value from passive data, overcoming the limitations of current correlation-based research.

#### Promoting Interdisciplinary Collaboration and Technological Implementation

The successful implementation of these technologies depends on deep interdisciplinary collaboration among clinical medicine, data science, engineering, ethics, and policy making [108]. It is recommended to form interdisciplinary alliances involving psychiatrists, data scientists, ethicists, and biomedical engineers to jointly establish technical standards and ethical guidelines (such as those from international organizations such as the International Medical Device Regulators Forum [124]). Strengthening strategic collaborations among governments, industry, and academia is also critical. Only with the dual support of policy and market forces can the technology be scaled and eventually translated into practical tools that enhance public health and improve patient outcomes.

#### Strengthening Cross-Cultural Validation and Inclusive Research

Future research should prioritize inclusive recruitment strategies such as collaborating with community health organizations, using multilingual sensing platforms, and validating models within low-income or rural populations. Causal frameworks for cross-cultural studies (eg, standardized data generation mechanisms and multigroup stratified analysis) provide a methodological foundation for cross-cultural validation in global digital phenotyping research. By systematically integrating cultural differences, these approaches can enhance the translational relevance of research findings across diverse populations [125].

### Conclusions

This study provides a comprehensive assessment of the current status, technical pathways, and future directions of passive sensing technologies based on wearable devices and smartphones combined with ML for mental health monitoring. We found that passive features such as sleep, physical activity, physiological signals, and social behavior showed stable associations with various mental disorders, including depression, anxiety, bipolar disorder, and schizophrenia. Among these features, heart rate, movement index, and step count were the most commonly used indicators. At the algorithmic level, deep learning models (eg, CNN and LSTM) demonstrated excellent performance in mental health prediction due to their robust temporal data processing capabilities. In contrast, traditional methods such as random forest and XGBoost continue to play a significant role in clinical research due to their strong interpretability.

Despite these advancements, challenges such as small sample sizes, short monitoring durations, limited device types, data noise and missing values, lack of model interpretability, and insufficient external validation hinder the generalizability and clinical translation of these studies. To advance the field, future research should focus on large-scale, multicenter, longitudinal studies across diverse populations; establish standardized protocols for data collection and preprocessing; strengthen multimodal integration and personalized modeling; and introduce explainable AI and privacy protection mechanisms to ensure the safe and efficient application of passive sensing technologies in real-world health care environments.

## Appendix

Multimedia Appendix 1 PRISMA-ScR checklist.

Multimedia Appendix 2 Search strategy.

Multimedia Appendix 3 Detailed definitions and classifications of behavioral categories.

Multimedia Appendix 4 Detailed information on the included studies.

## Abbreviations

AI: artificial intelligence
AUC: area under the curve
CNN: convolutional neural network
ECG: electrocardiography
KNN: k-nearest neighbor
LSTM: long short-term memory
MeSH: Medical Subject Headings
ML: machine learning
PRISMA: Preferred Reporting Items for Systematic Reviews and Meta-Analyses
PRISMA-ScR: Preferred Reporting Items for Systematic Reviews and Meta-Analyses extension for Scoping Reviews
SVM: support vector machine
XGBoost: Extreme Gradient Boosting

## Data Availability

The datasets generated or analyzed during this study are available from the corresponding author on reasonable request.
