# Supplementary material for: Passive Sensing for Mental Health Monitoring Using Machine Learning With Wearables and Smartphones: Scoping Review
Source: J Med Internet Res. 2025 Aug 14;27:e77066. doi: 10.2196/77066 (PMC12395114; doi:10.2196/77066)
Supplement: Multimedia Appendix 2 [file jmir_v27i1e77066_app2.docx]

| **Database** | **Search query** |
| --- | --- |
| **Web of Science Core Collection** | TS=（Wearable OR wear* OR smartwatch* OR smartphone* OR "cell phone*" OR "mobile application*" OR "digital application*" OR "smart phone" OR "personal device*" OR "digital data" OR "health app" OR monitor* OR "personal digital assistant" OR track* OR biosensor* OR biosensing OR sensor* OR sensing OR "mobile app"）AND TS=("Machine Learning" OR "Bayesian Learning" OR "Deep Learning" OR "Statistical Learning" OR "Artificial Intelligence" OR "Unsupervised Learning" OR "Supervised Learning" OR "Pattern Recognition" OR "Data Mining" OR "Cluster*" OR "Probability Learning" OR "Neural Network" OR "Support Vector Machine" OR "Random Forest" OR "Gradient Boosting") AND TS=("mental illness" OR "mental disorder*" OR "affective disorder*" OR "psychotic disorder*" OR "post-traumatic stress disorder*" OR "PTSD" OR distress OR "depress*" OR anxiety OR bipolar OR schizophrenia OR psychosis OR "mental health" OR "positive affect*" OR "negative affect*" OR "positive emotion*" OR "negative emotion*" OR mood OR "emotional regulation" OR "burnout" OR "suicidal ideation") |
| **Pubmed** | ("Wearable" OR "wear*" OR "smartwatch*" OR "smartphone*" OR "cell phone*" OR "mobile application*" OR "digital application*" OR "smart phone" OR "personal device*" OR "digital data" OR "health app" OR "monitor*" OR "personal digital assistant" OR "track*" OR "biosensor*" OR "biosensing" OR "sensor*" OR "sensing" OR "mobile app")  AND  ("Machine Learning" OR "Bayesian Learning" OR "Deep Learning" OR "Statistical Learning" OR "Artificial Intelligence" OR "Unsupervised Learning" OR "Supervised Learning" OR "Pattern Recognition" OR "Data Mining" OR "Cluster*" OR "Probability Learning" OR "Neural Network" OR "Support Vector Machine" OR "Random Forest" OR "Gradient Boosting")  AND  ("mental illness" OR "mental disorder*" OR "affective disorder*" OR "psychotic disorder*" OR "post-traumatic stress disorder*" OR "PTSD" OR "distress" OR "depress*" OR "anxiety" OR "bipolar" OR "schizophrenia" OR "psychosis" OR "mental health" OR "positive affect*" OR "negative affect*" OR "positive emotion*" OR "negative emotion*" OR "mood" OR "emotional regulation" OR "burnout" OR "suicidal ideation") |
| **IEEE Xplore** | (("Wearable" OR "wear*" OR "smartwatch" OR "smartwatches" OR "smartphone" OR "smartphones" OR "cell phone" OR "cell phones" OR "mobile application" OR "mobile applications" OR "digital application" OR "digital applications" OR "smart phone" OR "personal device*" OR "digital data" OR "health app" OR "monitor" OR "monitors" OR "personal digital assistant" OR "track" OR "tracking" OR "biosensor*" OR "biosensing" OR "sensor" OR "sensors" OR "sensing" OR "mobile app")  AND  ("Machine Learning" OR "Bayesian Learning" OR "Deep Learning" OR "Statistical Learning" OR "Artificial Intelligence" OR "Unsupervised Learning" OR "Supervised Learning" OR "Pattern Recognition" OR "Data Mining" OR "cluster" OR "clusters" OR "Probability Learning" OR "Neural Network" OR "Support Vector Machine" OR "Random Forest" OR "Gradient Boosting")  AND  ("mental illness" OR "mental disorder" OR "mental disorders" OR "affective disorder" OR "affective disorders" OR "psychotic disorder" OR "psychotic disorders" OR "post-traumatic stress disorder*" OR "PTSD" OR "distress" OR "depression" OR "depressive" OR "anxiety" OR "bipolar" OR "schizophrenia" OR "psychosis" OR "mental health" OR "positive affect*" OR "negative affect*" OR "positive emotion" OR "positive emotions" OR "negative emotion" OR "negative emotions" OR "mood" OR "emotional regulation" OR "burnout" OR "suicidal ideation")) |
| **Scoupes** | (TITLE-ABS-KEY (Wearable OR wear* OR smartwatch* OR smartphone* OR "cell phone*" OR "mobile application*" OR "digital application*" OR "smart phone" OR "personal device*" OR "digital data" OR "health app" OR monitor* OR "personal digital assistant" OR track* OR biosensor* OR biosensing OR sensor* OR sensing OR "mobile app"))  AND  (TITLE-ABS-KEY ("Machine Learning" OR "Bayesian Learning" OR "Deep Learning" OR "Statistical Learning" OR "Artificial Intelligence" OR "Unsupervised Learning" OR "Supervised Learning" OR "Pattern Recognition" OR "Data Mining" OR "Cluster*" OR "Probability Learning" OR "Neural Network" OR "Support Vector Machine" OR "Random Forest" OR "Gradient Boosting"))  AND  (TITLE-ABS-KEY ("mental illness" OR "mental disorder*" OR "affective disorder*" OR "psychotic disorder*" OR "post-traumatic stress disorder*" OR "PTSD" OR distress OR "depress*" OR anxiety OR bipolar OR schizophrenia OR psychosis OR "mental health" OR "positive affect*" OR "negative affect*" OR "positive emotion*" OR "negative emotion*" OR mood OR "emotional regulation" OR "burnout" OR "suicidal ideation")) |
| **Embase** | ('wearable' OR 'wear*' OR 'smartwatch*' OR 'smartphone*' OR 'cell phone*' OR 'mobile application*' OR 'digital application*' OR 'smart phone' OR 'personal device*' OR 'digital data' OR 'health app' OR 'monitor*' OR 'personal digital assistant' OR 'track*' OR 'biosensor*' OR 'biosensing' OR 'sensor*' OR 'sensing' OR 'mobile app') AND  ('machine learning' OR 'bayesian learning' OR 'deep learning' OR 'statistical learning' OR 'artificial intelligence' OR 'unsupervised learning' OR 'supervised learning' OR 'pattern recognition' OR 'data mining' OR 'cluster*' OR 'probability learning' OR 'neural network' OR 'support vector machine' OR 'random forest' OR 'gradient boosting') AND  ('mental illness' OR 'mental disorder*' OR 'affective disorder*' OR 'psychotic disorder*' OR 'post-traumatic stress disorder*' OR 'PTSD' OR 'distress' OR 'depress*' OR 'anxiety' OR 'bipolar' OR 'schizophrenia' OR 'psychosis' OR 'mental health' OR 'mental wellness' OR 'wellbeing' OR 'well-being' OR 'SWB' OR 'happiness' OR 'happy' OR 'positive affect*' OR 'negative affect*' OR 'positive emotion*' OR 'negative emotion*' OR 'mood' OR 'life satisfaction' OR 'satisfaction with life' OR 'emotional regulation' OR 'burnout' OR 'suicidal ideation') |
| **PsycINFO** | (Wearable OR wear* OR smartwatch* OR smartphone* OR "cell phone*" OR "mobile application*" OR "digital application*" OR "smart phone" OR "personal device*" OR "digital data" OR "health app" OR monitor* OR "personal digital assistant" OR track* OR biosensor* OR biosensing OR sensor* OR sensing OR "mobile app")  AND ("Machine Learning" OR "Bayesian Learning" OR "Deep Learning" OR "Statistical Learning" OR "Artificial Intelligence" OR "Unsupervised Learning" OR "Supervised Learning" OR "Pattern Recognition" OR "Data Mining" OR Cluster* OR "Probability Learning" OR "Neural Network" OR "Support Vector Machine" OR "Random Forest" OR "Gradient Boosting")  AND ("mental illness" OR "mental disorder*" OR "affective disorder*" OR "psychotic disorder*" OR "post-traumatic stress disorder*" OR PTSD OR distress OR depress* OR anxiety OR bipolar OR schizophrenia OR psychosis OR "mental health" OR "positive affect*" OR "negative affect*" OR "positive emotion*" OR "negative emotion*" OR mood OR "emotional regulation" OR burnout OR "suicidal ideation") |
| **ACM Digital Library** | ("Wearable" OR "wear*" OR "smartwatch*" OR "smartphone*" OR "cell phone*" OR "mobile application*" OR "digital application*" OR "smart phone" OR "personal device*" OR "digital data" OR "health app" OR "monitor*" OR "personal digital assistant" OR "track*" OR "biosensor*" OR "biosensing" OR "sensor*" OR "sensing" OR "mobile app")  AND  ("Machine Learning" OR "Bayesian Learning" OR "Deep Learning" OR "Statistical Learning" OR "Artificial Intelligence" OR "Unsupervised Learning" OR "Supervised Learning" OR "Pattern Recognition" OR "Data Mining" OR "Cluster*" OR "Probability Learning" OR "Neural Network" OR "Support Vector Machine" OR "Random Forest" OR "Gradient Boosting")  AND  ("mental illness" OR "mental disorder*" OR "affective disorder*" OR "psychotic disorder*" OR "post-traumatic stress disorder*" OR "PTSD" OR "distress" OR "depress*" OR "anxiety" OR "bipolar" OR "schizophrenia" OR "psychosis" OR "mental health" OR "mental wellness" OR "wellbeing" OR "well-being" OR "SWB" OR "happiness" OR "happy" OR "positive affect*" OR "negative affect*" OR "positive emotion*" OR "negative emotion*" OR "mood" OR "life satisfaction" OR "satisfaction with life" OR "emotional regulation" OR "burnout" OR "suicidal ideation") |
