# Supplementary material for: Passive Sensing for Mental Health Monitoring Using Machine Learning With Wearables and Smartphones: Scoping Review
Source: J Med Internet Res. 2025 Aug 14;27:e77066. doi: 10.2196/77066 (PMC12395114; doi:10.2196/77066)
Supplement: Multimedia Appendix 3 [file jmir_v27i1e77066_app3.docx]

**Feature Descriptions for Sleep features extracted in all included studies.**

| **Features** | **Description** | **Study-level feature examples** | **Number of times used** |
| --- | --- | --- | --- |
| Total sleep Time | Actual sleep time: sleep episode duration minus wakefulness periods | Total sleep Time (TST),  Cumulative Sleep Time,  Overall Sleep Time | Anxiety(n=3)  Depression(n=8)  Bipolar Disorder（n=1）  Schizophrenia (n=2) |
| Sleep start time | The timestamp marking the beginning of a sleep episode | Sleep begin Time,  Sleep onset time | Schizophrenia（n=2）  Depression(n=2) |
| Sleep end time | The timestamp marking the conclusion of a sleep episode, excluding subsequent wakefulness. | Sleep offset,  Wake-up time | Schizophrenia(n=2)  Depression（n=5）  Anxiety(n=1) |
| Sleep Efficiency | The percentage of a sleep episode spent asleep, calculated as actual sleep time divided by total sleep episode duration | Sleep Efficiency,  Sleep efficiency score | Depression(n=5)  Anxiety（n=2）  Bipolar Disorder（n=1） |
| Number of Awakenings | The total count of discrete periods of wakefulness occurring during a sleep episode, with each awakening defined as a sustained interruption meeting a predetermined duration threshold. | Number of Awakenings,  Wake count during sleep,  Awakenings count | Depression（n=3）  Anxiety（n=1）  Bipolar Disorder（n=1） |
| Sleep midpoint | the point in time that lies exactly halfway between the sleep start and sleep end times, offering a measure of the central timing of the sleep episode. | Median bedtime | Bipolar Disorder （n=1）  Depression （n=1） |
| Time in bed | The total duration spent in bed, including both sleep time and wakefulness periods, from the moment the person gets into bed to when they get up. | Time spent in bed,  Bedtime duration | Depression （n=3）  Anxiety（n=1） |
| Sleep onset latency | The period from when a person goes to bed to the onset of sleep. It measures how quickly one falls asleep | Sleep Onset Latency (SOL),  Mean sleep latency | Depression（n=2）  Anxiety（n=2） |
| Sleep stages | The distinct phases of sleep (e.g., light sleep, deep sleep, and REM sleep) that occur cyclically during a sleep episode | Deep sleep count,  REM sleep count,  REM percent | Depression（n=3）  Bipolar Disorder（n=2） |
| Restless Counts | A tally of the number of movements or disturbances during sleep, indicating periods of restlessness | Number of Restless Episodes | Depression（N=2） |
| Duration of awakenings | The total time spent awake during the sleep period after initially falling asleep | Average awakening duration, Wake after sleep onset | Bipolar Disorder（N=1）  Depression（N=2）  Anxiety(N=2) |
| Percentage of wake time | The proportion of the sleep episode during which the person is awake, typically calculated relative to total time in bed | Wake duration percentage | Anxiety（N=1）  Bipolar Disorder（n=1） |
| Maximum activity during sleep | The highest level of physical movement or activity recorded during sleep, often reflecting disturbances or transitions between sleep stages | Peak activity during sleep | Anxiety（N=1）  Bipolar Disorder（N=1） |
| Time dozing before rising | The interval of light sleep or drowsiness immediately preceding full wakefulness, marking the gradual transition from sleep to being fully awake | Dozing time before getting up | Anxiety（N=2）  Depression（N=1） |

**Feature descriptions for Environmental features extracted in all included studies.**

| **Features** | **Description** | **Study-level feature examples** | **Number of times used** |
| --- | --- | --- | --- |
| EDA/skin conductance | Changes in skin’s electrical conductivity due to sweat | Electrodermal activity, Skin conductance | Bipolar Disorder (n=2)  Depression (n=3)  Schizophrenia (n=1) |
| Skin temperature | The temperature of the skin | Average Skin Temperature, Local Temperature Gradient | Bipolar Disorder (n=2)  Depression (n=4)  Schizophrenia (n=1) |
| Heart rate | The number of heartbeats per minute | Resting Heart Rate,  Average Heart Rate,  Maximum Heart Rate,  Heart Rate Trends | Bipolar Disorder (n=3)  Depression (n=20)  Anxiety (n=5) |
| Heart rate variability | Variation in intervals between heartbeats, reflecting autonomic balance | Standard Deviation of NN intervals,  Low Frequency,  Root Mean Square of Successive Differences | Depression （n=6）  Anxiety （n=2）  Schizophrenia （n=1）  Attention Deficit Hyperactivity Disorder （n=1）  Post-Traumatic Stress Disorder （n=1） |

**Feature Descriptions for Physical Activity features extracted in all included studies.**

| **Features** | **Description** | **Study-level feature examples** | **Number of times used** |
| --- | --- | --- | --- |
| Mean acceleration/activity count | The average value of acceleration or activity counts during the monitoring period | Average acceleration, Mean vector magnitude, Movement count, Activity level count | Post-Traumatic Stress Disorder (n=1)  Depression （n=1）Schizophrenia （n=2）Anxiety （n=2） |
| Total acceleration/ activity count | The sum of all recorded acceleration or activity counts during the monitoring period | Magnitude of acceleration, Activity counts | Schizophrenia (n=1)  Depression (n=1)  Anxiety (n=1) |
| Duration of still or sedentary periods | The total time spent in a still or low-activity state during the monitoring period | Sedentary time duration, Longest still period | Bipolar Disorder （n=1）  Depression （n=2） |
| Distance walked on foot | The total distance walked | Walking distance, Distance traveled by walking | Depression (n=2)  Suicidal Ideation （n=1） |
| Step count | The total number of steps recorded | Total Steps, Walking Steps | Bipolar Disorder （n=1）  Depression (n=14)  Suicidal Ideation （n=1）  Anxiety （n=1） |
| Duration of light activity periods | The cumulative time spent in low-intensity activity, defined by pre-set acceleration thresholds | Light activity duration, Light activity start times | Depression (n=3)  Anxiety （n=1） |
| Duration of moderate activity periods | The cumulative time spent in moderate-intensity activity, defined by acceleration data and activity thresholds | Moderate activity duration, Total moderate activity time | Depression (n=3)  Anxiety (n=1) |
| Duration of vigorous activity periods | The cumulative time spent in high-intensity activity, as determined by accelerometer data | Vigorous activity time, Average duration per vigorous bout, Frequency of vigorous activity periods | Depression (n=3)  Anxiety （n=1） |
| Movement index | A composite index constructed from multiple accelerometer data points, aiming to reflect an individual’s overall activity level. | Amplitude, Activity Intensity, Activity Level | Schizophrenia （n=4）Depression (n=15)  Bipolar Disorder （n=2）  Post-Traumatic Stress Disorder （n=1）  Anxiety （n=2）  Attention Deficit Hyperactivity Disorder （n=1） |
| Energy expenditure | The estimated energy expenditure based on physical activity monitoring data and metabolic models, typically measured in kilocalories (kcal) | Caloric expenditure | Depression （n=3） |
| Exercise Cycle | Describes the periodicity or regularity of an individual’s physical activity, reflecting the temporal characteristics of exercise behavior | Frequency of cycles, Activity cycle | Depression (n=3)  Schizophrenia (n=2)  Anxiety （n=1） |

**Feature descriptions for Phone Use features extracted in all included studies.**

| **Features** | **Description** | **Study-level feature examples** | **Number of times used** |
| --- | --- | --- | --- |
| Phone use during sleep | Refers to the usage of the phone during sleep, measuring the frequency and duration of phone interactions while sleeping | Phone use during sleep | Depression (n=1) |
| App usage | Refers to the usage of various mobile applications, including frequency and duration | Software usage, App open intervals, Device screen on time during app use | Schizophrenia (n=2)  Depression (n=4)  Suicidal Ideation (n=1)  Anxiety (n=1) |
| Screen event count/duration | Refers to the total number of events and the duration of each event related to changes in the device’s screen state, including turning the screen on, turning it off, locking, and unlocking. | Screen unlock count, Active screen time, Screen usage duration | Schizophrenia (n=1)  Depression (n=7) |

**Feature Descriptions for Circadian Rhythm features extracted in all included studies.**

| **Features** | **Description** | **Study-level feature examples** | **Number of times used** |
| --- | --- | --- | --- |
| Physical activity | Reflects the user's activity level at different times of the day, usually showing more activity during the day and reduced movement at night, illustrating the regular patterns of daily activity | Duration of activity periods and their day-night variation, Distribution and duration of inactive or resting periods | Bipolar Disorder （n=2）  Anxiety （n=1）  Depression (n=3) |
| sleep | Indicates the duration of sleep as well as the times of falling asleep and waking up, demonstrating the basic patterns of rest and activity in daily life | interdaily stability, intradaily variability of sleep-wake patterns | Depression (n=3)  Post-Traumatic Stress Disorder (n=1) |
| Phone use | Reflects the frequency and duration of the user's interactions with their phone throughout the day, typically exhibiting a periodic variation that reveals usage habits at different times | Distribution of unlock events across 24 hours | Depression （n=1）  Schizophrenia (n=1) |
| Sociability | The frequency and timing of social interactions generally follow daily patterns, with more interactions during the day and fewer at night, reflecting common behavioral trends | Frequency of social interactions across different times of day | Depression （n=1） |
| Physiology | Physiological indicators such as body temperature, heart rate, and hormone secretion typically change in a regular manner over time, reflecting the inherent daily rhythm of the human body | Continuous or periodic heart rate measurements over 24 hours | Depression （n=1） |

**Feature descriptions for Environmental features extracted in all included studies.**

| **Features** | **Description** | **Study-level feature examples** | **Number of times used** |
| --- | --- | --- | --- |
| Humidity | Humidity | Humidity | Depression (n=1)  Anxiety (n=1) |
| Ambient Light | Ambient Light | Ambient Light | Depression (n=3)  Anxiety （n=1）  Schizophrenia (n=1)  Suicidal Ideation (n=1) |
| Ambient sound | Ambient sound | Ambient sound | Schizophrenia (n=1)  Anxiety （n=1） |
| UV Exposure | UV Exposure | UV Exposure | Depression (n=1) |
| Precipitation | Precipitation | Precipitation | Depression (n=1)  Anxiety （n=1） |

**Feature descriptions for Location features extracted in all included studies.**

| **Features** | **Description** | **Study-level feature examples** | **Number of times used** |
| --- | --- | --- | --- |
| Total distance | The cumulative distance traveled within a specific time period | Movement distance, Cumulative distance | Schizophrenia （n=1）  Depression （n=3）  Suicidal Ideation （n=1） |
| Time spent at home | The total duration of time a user spends at home | Stationary time at home, Time stayed at home | Suicidal Ideation （n=1）  Depression （n=4）  Anxiety （n=1） |
| Location entropy | A measure of the diversity and uncertainty of visited locations | Location entropy | Anxiety （n=2）  Bipolar Disorder （n=1）  Depression （n=4）  Schizophrenia （n=1） |
| Normalized location entropy | The location entropy scaled to a fixed range | Normalized location entropy | Depression （n=2） |
| Location variance | The degree of dispersion in the geographic distribution of locations | Location variance | Depression （n=2） |
| Transition time | The total time spent moving between different locations | Travel time between locations | Depression （n=3）  Anxiety （n=1） |
| Transition frequency | The number of location transitions per unit time | Transition frequency | Anxiety （n=1）  Depression （n=1） |
| Average moving speed | The average speed calculated during movement | Average moving speed | Depression （n=1） |
| Number of unique or significant locations | The count of different locations visited over a period of time | Number of distinct locations, Total visited locations | Depression （n=4）  Anxiety （n=2）  Schizophrenia （n=1） |
| distance from home | The distance between the user's location during outings and their home | distance from home | Depression （n=1） |
| Time at location | The duration of time spent at a specific location | Duration at location | Schizophrenia （n=1）  Depression （n=1） |

**Feature descriptions for Sociability features extracted in all included studies.**

| **Features** | **Description** | **Study-level feature examples** | **Number of times used** |
| --- | --- | --- | --- |
| Number of incoming/outgoing calls | The total count of incoming and outgoing calls over a specified period | Incoming Calls Count, Outgoing Calls Count, Unique Contacts Received, Unique Contacts Called | Depression (n=9)  Anxiety (n=1)  Schizophrenia (n=2)  Bipolar Disorder (n=1) |
| Duration of incoming/outgoing calls | The cumulative time spent on incoming and outgoing calls | Incoming call duration, Outgoing call duration, Call length、Call duration | Schizophrenia (n=1)  Bipolar Disorder (n=1)  Anxiety (n=1)  Suicidal Ideation (n=1)  Depression (n=7) |
| Text message timestamp | The recorded time at which text messages are sent or received | Text message sent timestamp, Text message received timestamp, Last message timestamp | Depression (n=6) |
| Socialisation By Proximity | A measure of social interaction based on physical proximity to others | Socialisation By Proximity | Depression (n=1) |
| Number of missed calls | The total count of calls that were not answered | Missed call count, Missed call number | Depression (n=2)  Anxiety (n=2) |
| Calling entropy | An indicator of the variability or unpredictability in calling behavior | Calling entropy | Depression (n=2)  Anxiety （n=1） |
| Length of incoming/outgoing text messages | The average or total length (in characters) of text messages exchanged | Incoming text message length | Depression (n=2) |
| Number of incoming/outgoing text messages | The total count of text messages sent and received | Number of incoming text messages, Total number of text messages, Sent message count | Depression (n=5)  Schizophrenia (n=1) |
| Conversation Frequency | The number of distinct conversational events within a given period | Number of Conversations, Access Frequency | Depression (n=3) |
| Unique Remote Interactions | Total number of unique individuals with whom a participant interacted through phone calls or SMS messages on a particular day | Number of Unique, Remote Interactions | Anxiety (n=2)  Bipolar Disorder (n=1)  Depression (n=5) |

**Glossary of terms**

**Accelerometer** — An electronic sensor that measures the acceleration forces acting on an object, allowing for detection of movement, orientation, and dynamic changes in position. It typically operates based on capacitive, piezoelectric, or resistive principles and is widely used in activity monitoring, motion analysis, and navigation applications.

**Skin Temperature Sensor** — A device that measures the surface temperature of the skin, typically using thermistors or thermocouples. It provides non-invasive monitoring of body temperature variations, which can be useful in health assessment, fever detection, and physiological studies.

**Pedometer** — A device that passively detects and records steps or movement counts, typically using mechanical switches or accelerometers.

**GPS (Global Positioning System)** — A satellite-based system that determines geographic location, velocity, and time by processing signals received from a constellation of satellites orbiting the Earth.

**App usage** — The collection of data related to the activation, duration, and frequency of mobile applications on a device, typically gathered passively through software logs or monitoring tools.

**Bluetooth** — A short-range wireless communication technology that enables data exchange between devices over short distances using ultra-high frequency (UHF) radio waves.

**WiFi Sensor** — A device that detects and utilizes wireless signals from WiFi networks to determine location, connectivity status, or generate data based on signal strength and network presence.

**Screen** — A display device that visually presents information and can passively record interaction patterns such as screen-on duration, usage frequency, and user engagement metrics.

**Call logs** — A record of incoming, outgoing, and missed phone calls, including timestamps and duration, used to passively collect communication activity data.

**UV Light Sensor** — A device that passively detects ultraviolet light levels in the environment by measuring the intensity of UV radiation.

**Humidity Sensor** — A device that passively measures the humidity level of the surrounding environment by detecting moisture content in the air.

**Microphone** — A device that passively detects sound waves and converts them into electrical signals, used to record environmental audio and acoustic activity.

**Light Sensor** — A device that passively detects ambient light levels by measuring the intensity of light in the environment, often used to monitor lighting conditions or infer activity patterns.

**PPG (Photoplethysmography)** — A non-invasive optical sensor that passively detects blood volume changes in the microvascular bed of tissue by measuring light absorption or reflection.

**Magnetometer** — A sensor that passively measures the strength and direction of magnetic fields, often used to determine orientation relative to the Earth's magnetic field.

**SMS logs** — Records of sent and received text messages, including timestamps and contact information, used to passively collect communication activity data.

**Electrodermal activity** — The passive measurement of skin conductance changes caused by sweat gland activity, indicating physiological arousal.

**Pressure Sensor** — A device that passively detects and measures external or internal pressure by converting the force exerted on it into an electrical signal.

**Gyroscope** — A device that passively measures angular velocity and orientation changes by detecting rotational motion around its axes.

**Heart Rate Sensor** — A device that passively detects and measures the number of heartbeats per minute, typically using optical (e.g., PPG) or electrical (e.g., ECG) methods.

**EEG (Electroencephalography)** — A passive recording technique that detects electrical activity generated by neural oscillations in the brain via electrodes placed on the scalp.
